# Osteolectin increases bone elongation and body length by promoting growth plate chondrocyte proliferation

**DOI:** 10.1073/pnas.2220159120

**Published:** 2023-05-22

**Authors:** Jingzhu Zhang, Liming Du, Bethany Davis, Zhimin Gu, Junhua Lyu, Zhiyu Zhao, Jian Xu, Sean J. Morrison

**Affiliations:** ^a^Children’s Research Institute, University of Texas Southwestern Medical Center, Dallas, TX 75390; ^b^Department of Pediatrics, University of Texas Southwestern Medical Center, Dallas, TX 75390; ^c^HHMI, University of Texas Southwestern Medical Center, Dallas, TX 75390

**Keywords:** osteolectin, integrin α11, chondrocyte, bone elongation, osteogenesis

## Abstract

Osteolectin is a recently identified osteogenic growth factor that binds to Integrin α11 (encoded by *Itga11*), promoting Wnt pathway activation and osteogenic differentiation by bone marrow stromal cells. While *Osteolectin* and *Itga11* are not required for the formation of the skeleton during fetal development, they are required for the maintenance of adult bone mass. Genome-wide association studies in humans reported a single-nucleotide variant (rs182722517) 16 kb downstream of *Osteolectin* associated with reduced height and plasma Osteolectin levels. In this study, we tested whether Osteolectin promotes bone elongation and found that *Osteolectin*-deficient mice have shorter bones than those of sex-matched littermate controls. Integrin α11 deficiency in limb mesenchymal progenitors or chondrocytes reduced growth plate chondrocyte proliferation and bone elongation. Recombinant Osteolectin injections increased femur length in juvenile mice. Human bone marrow stromal cells edited to contain the rs182722517 variant produced less Osteolectin and underwent less osteogenic differentiation than that of control cells. These studies identify Osteolectin/Integrin α11 as a regulator of bone elongation and body length in mice and humans.

## Significance

Osteolectin promotes the maintenance of bone mass in adult mice, but there has not been genetic evidence that Osteolectin is functionally important in humans. In the current study, we identified a function for Osteolectin in juvenile mice: the promotion of bone elongation by increasing growth plate chondrocyte proliferation, leading to increased body length. Second, we describe a human genetic variant that suggests Osteolectin also promotes bone elongation in humans. The single-nucleotide variant (rs182722517) that is associated with reduced height and plasma Osteolectin levels in humans is also associated with reduced Osteolectin expression in bone marrow stromal cells.

Height is a polygenic trait (1) determined by the longitudinal growth of limb bones and vertebrae. In juvenile mammals, bones grow longitudinally as a result of the proliferation and osteogenic differentiation of chondrocytes in the growth plate (2). Chondrocyte proliferation and bone growth are systemically promoted by hormones (3) as well as by the activation of Wnt (4, 5) and Hedgehog (6, 7) signaling in chondrocytes.

In young mice, skeletal stem cells are present among parathyroid hormone (PTH)–related protein (Pthrp)-CreER and Aggregan-expressing chondrocytes in the resting zone of the growth plate (8). These stem cells proliferate to form columns of chondrocytes in the growth plate that then differentiate into osteoblasts that contribute to bone growth (8). The chondrocytes also give rise to leptin receptor–expressing (LepR^+^) stromal cells that migrate into the bone marrow metaphysis (8, 9). Dlx-CreER^+^ perichondrial cells contribute to the formation of diaphyseal bone during fetal and early postnatal development and give rise to LepR^+^ cells in diaphysis bone marrow (10). The LepR^+^ cells that arise from these sources are rare in early postnatal bone marrow but expand in number to account for 0.3% of cells in adult bone marrow, where they are a key source of growth factors for the maintenance of hematopoietic stem cells and restricted hematopoietic progenitors (11–14). The LepR^+^ cells also include the skeletal stem cells that are the main source of osteoblasts and adipocytes in adult bone marrow (15), as well as restricted osteogenic (16) and adipogenic (17) progenitors.

Osteolectin (Clec11a) is an osteogenic growth factor that binds to Integrin α11 (encoded by *Itga11*), promoting Wnt pathway activation and osteogenic differentiation by bone marrow stromal cells, including LepR^+^ cells (18, 19). Osteolectin and Integrin α11 are not necessary for the regulation of hematopoiesis or the formation of the skeleton during fetal or early postnatal development but are necessary for the maintenance of adult bone mass, at least in mice (18–20). Osteolectin is synthesized by LepR^+^ bone marrow stromal cells, hypertrophic chondrocytes, osteoblasts, osteocytes, and periosteal cells (16, 18, 19).

Human and mouse Osteolectin are 80% identical at the amino acid level. Recombinant human Osteolectin promotes osteogenic differentiation by human bone marrow stromal cells (18, 19). Osteolectin expression is induced by PTH in mice and humans, and it mediates the osteogenic effects of PTH in mice (20); however, there is not yet functional evidence that Osteolectin regulates the human skeleton in vivo.

Osteolectin and Integrin α11 were not previously known to play any role in the regulation of bone elongation or height/body length. However, when we analyzed the results from genome-wide association studies of human genetic variants associated with differences in plasma protein levels (21) and height (22–24), we found a single-nucleotide variant (rs182722517) 16 kb downstream of *Osteolectin* that is associated with reduced plasma Osteolectin levels and height. Based on these observations, we set out to test whether Osteolectin promotes bone elongation.

## Results

### Osteolectin Promotes Chondrocyte Proliferation and Bone Elongation.

To test whether Osteolectin regulates longitudinal bone growth, *Osteolectin*-deficient mice (18) were analyzed at postnatal day 4 (P4), P14, 4 week (4W), and 8 week (8W) of age. At P4 and P14, *Osteolectin*-deficient and sex-matched littermate control mice exhibited no differences in body mass, the lengths of femurs or the third lumbar spine (LS3) vertebrae (*SI Appendix*, Fig. S1 *A* and *B*), or femur or vertebra bone parameters (*SI Appendix*, Fig. S1 *C*–*H*). *Osteolectin* deficiency thus had no effect on the formation or growth of the skeleton prior to P14.

**Fig. 1.**
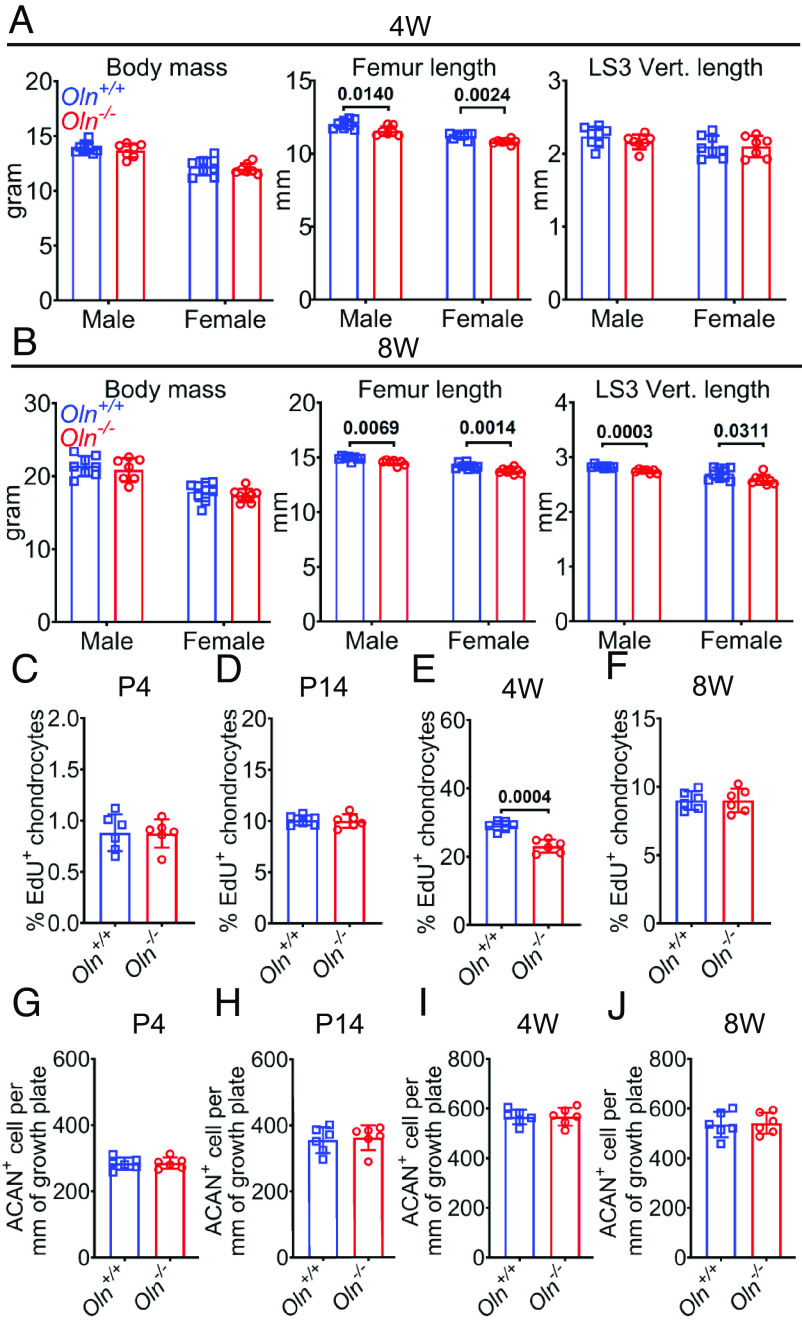
Osteolectin promoted growth plate chondrocyte proliferation and longitudinal bone growth in juvenile mice. (*A* and *B*) Body mass, femur length, and LS3 vertebra length in *Osteolectin-*deficient (*Oln^−/−^*) and sex-matched littermate control (*Oln^+/+^*) mice at 4 week (4W, *A*) and 8 week of age (8W, *B*). Each square/circle represents a different mouse (seven to nine mice per sex per age per genotype in five or six independent experiments per age). (*C*–*F*) The percentages of Aggrecan^+^ growth plate chondrocytes that were EdU^+^ at P4 (*C*), P14 (*D*), 4W (*E*), and 8W (*F*) of age. (*G*–*J*) Numbers of Aggrecan^+^ chondrocytes per mm of growth plate in *Oln^−/−^* and sex-matched littermate control (*Oln^+/+^*) mice at P4 (*G*), P14 (*H*), 4W (*I*), and 8W (*J*) of age (Three mice per sex per genotype per age in three independent experiments per age for panels *C*–*J*). All statistical tests were two sided. All data represent mean ± SD. Statistical significance was assessed using Student’s *t* tests followed by Holm–Sidak’s multiple comparisons test (femur length in *A* and LS3 vertebra length in *B*, and *C*–*J*), or two-way ANOVAs followed by Sidak’s multiple comparisons test (other panels in *A* and *B*).

At 4 and 8 wk of age, *Osteolectin*-deficient and sex-matched littermate control mice did not differ in body mass (Fig. 1 *A* and *B*) or in femur cortical bone parameters (*SI Appendix*, Fig. S2 *A* and *B*). At 4 wk of age, *Osteolectin*-deficient and control mice also did not differ in trabecular bone parameters within femurs or vertebrae (*SI Appendix*, Fig. S2 *C* and *D*) or vertebral length (Fig. 1*A*), but *Osteolectin*-deficient mice had femurs that were 3 to 4% shorter than those in control mice (Fig. 1*A*). At 8 wk of age, the *Osteolectin*-deficient mice had femurs and vertebrae that were 3 to 4% shorter than those of control mice (Fig. 1*B*) as well as reduced trabecular bone volume, number, and thickness (*SI Appendix*, Fig. S2 *E* and *F*). Thus, Osteolectin is required for bone elongation in juvenile mice, even before detectably contributing to trabecular bone volume.

To test whether Osteolectin promotes growth plate chondrocyte proliferation, we administered a 4-h to 2-d pulse (depending on mouse age) of 5-ethynyl-2′-deoxyuridine (EdU) to *Osteolectin*-deficient and sex-matched littermate control mice to mark dividing chondrocytes. At P4, P14, and 8 wk of age, we observed no difference in the percentage of Aggrecan^+^ chondrocytes in the growth plate that incorporated EdU (*SI Appendix*, Fig. S1*I* and Fig. 1 *C*, *D*, and *F*). However, at 4 wk of age, significantly fewer growth plate chondrocytes were EdU^+^ in *Osteolectin*-deficient as compared to control mice (*SI Appendix*, Fig. S1*I* and Fig. 1*E*). Cell death appeared to be rare in growth plate chondrocytes at all stages as we were unable to find any TUNEL^+^ cells (25) in the growth plates of either *Osteolectin*-deficient or control mice (*SI Appendix*, Fig. S2 *G* and *H*). At all stages (P4, P14, 4 wk, and 8 wk), the number of Aggrecan^+^ chondrocytes per millimeter of growth plate was similar in *Osteolectin*-deficient and sex-matched littermate control mice (*SI Appendix*, Fig. S1*I* and Fig. 1 *G*–*J*). Since growth plate chondrocytes differentiate into osteoblasts (26, 27), this raised the possibility that the decrease in chondrocyte proliferation in *Osteolectin*-deficient mice at 4 wk of age led to reduced osteogenesis and reduced bone elongation.

### Integrin α11 Promotes Chondrocyte Proliferation and Bone Elongation.

We tested whether the Osteolectin receptor, Integrin α11 (encoded by *Itga11*), regulates bone elongation by conditionally deleting *Itga11* (19) using *Prx1^Cre^*. *Prx1^Cre^* recombines during fetal development in limb mesenchymal cells but not in axial skeleton cells (including vertebrae) (28). We compared femur bone parameters between *Prx1^Cre^;Itga11^fl/fl^* mice and sex-matched *Itga11^fl/fl^* littermate controls at P4 and P14. *Prx1^Cre^;Itga11^fl/fl^* and *Itga11^fl/fl^* controls did not differ in body mass or femur length (*SI Appendix*, Fig. S3 *A* and *B*), or femur cortical (*SI Appendix*, Fig. S3 *C* and *D*) or trabecular bone parameters (*SI Appendix*, Fig. S3 *E* and *F*). *Itga11* deficiency thus had no effect on the formation or growth of limb bones prior to P14.

**Fig. 2.**
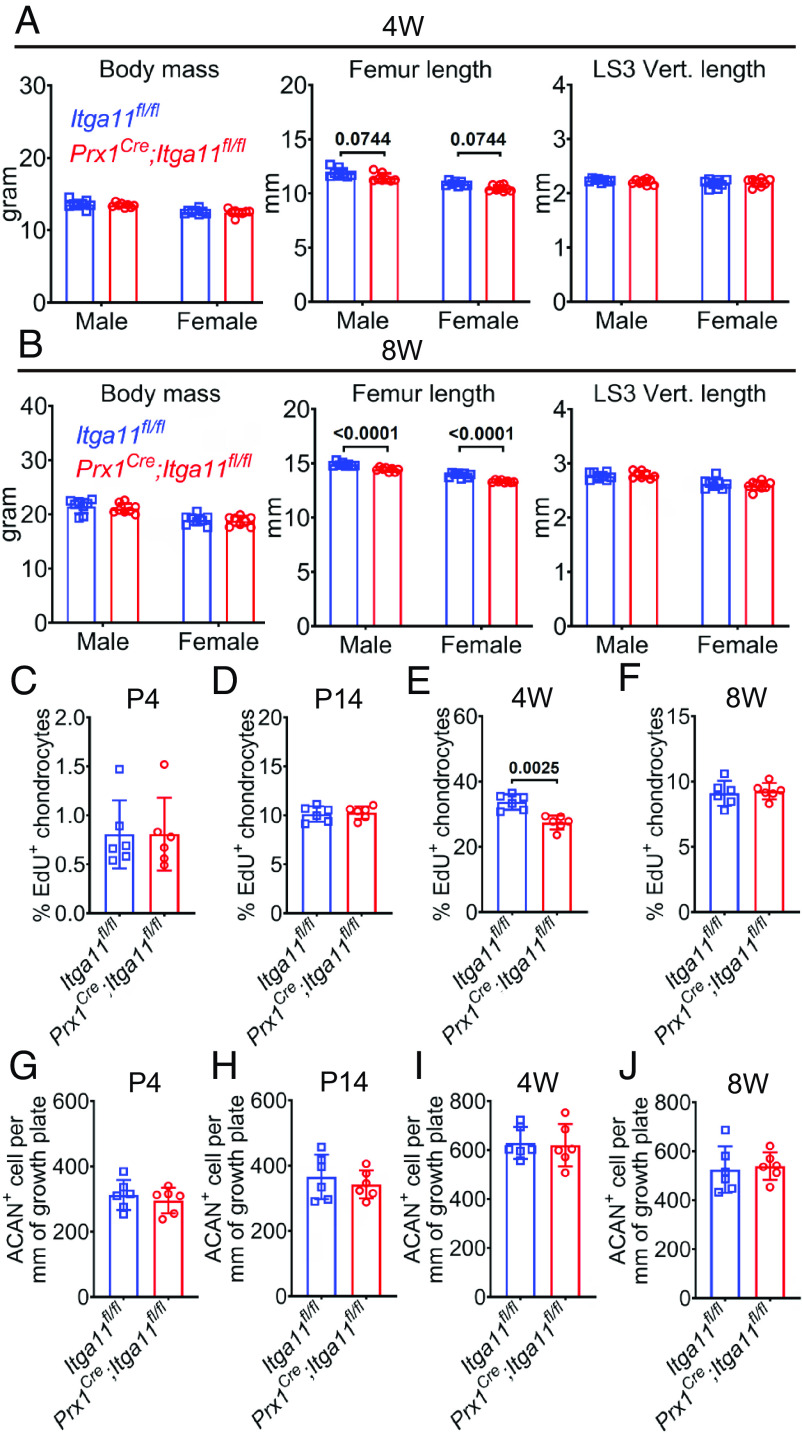
Integrin α11 promoted growth plate chondrocyte proliferation and longitudinal bone growth in juvenile mice. (*A* and *B*) Body mass, femur length, and LS3 vertebra length in *Prx1^Cre^;Itga11^fl/fl^* and sex-matched littermate control (*Itga11^fl/fl^*) mice at 4 wk (*A*) and 8 wk (*B*) of age (Eight mice per sex per age per genotype in five independent experiments per age). (*C*–*J*) *Prx1^Cre^;Itga11^fl/fl^* and littermate control (*Itga11^fl/fl^*) mice were administered pulses of EdU for 4 h (P4), 1 d (P14), or 2 d (4W and 8W). The percentages of Aggrecan^+^ growth plate chondrocytes that were EdU^+^ at P4 (*C*), P14 (*D*), 4W (*E*), and 8W (*F*) of age. Numbers of Aggrecan^+^ chondrocytes per mm of growth plate in *Prx1^Cre^;Itga11^fl/fl^* and sex-matched littermate control (*Itga11^fl/fl^*) mice at P4 (*G*), P14 (*H*), 4W (*I*), and 8W (*J*) of age (Three mice per sex per genotype per age in three independent experiments per age for panels *C*–*J*). All statistical tests were two sided. All data represent mean ± SD. Statistical significance was assessed using Mann–Whitney tests followed by Holm–Sidak’s multiple comparisons test (femur length in *A*), or two-way ANOVAs followed by Sidak’s multiple comparison tests (*B* and body mass in *A*), or Student’s *t* tests followed by Holm–Sidak’s multiple comparison tests (LS3 vertebra length in *A* and *C*–*J*).

At 4 and 8 wk of age, *Prx1^Cre^;Itga11^fl/fl^* mice and sex-matched *Itga11^fl/fl^* littermate controls did not differ in body mass or vertebra length (Fig. 2 *A* and *B*) or femur cortical bone parameters (*SI Appendix*, Fig. S4 *A* and *B*); however, at 4 wk of age, there was a trend toward shorter femurs in *Prx1^Cre^;Itga11^fl/fl^* mice (Fig. 2*A*) and at 8 wk of age, femurs were significantly (3 to 4%) shorter in *Prx1^Cre^;Itga11^fl/fl^* as compared to control mice (Fig. 2*B*). At 4 wk of age, we observed no differences in femur trabecular bone parameters (*SI Appendix*, Fig. S4*C*) but at 8 wk of age, we observed significantly reduced femur trabecular bone volume, number, and thickness in *Prx1^Cre^;Itga11^fl/fl^* as compared to control mice (*SI Appendix*, Fig. S4*D*). Therefore, Integrin α11 is also required for bone elongation in juvenile mice.

To test whether Integrin α11 promotes growth plate chondrocyte proliferation, we administered a 4-h to 2-d pulse of EdU to *Prx1^Cre^;Itga11^fl/fl^* and sex-matched littermate control mice. At P4, P14, and 8 wk of age, we observed no difference in the percentage of Aggrecan^+^ growth plate chondrocytes that incorporated EdU (Fig. 2 *C*, *D*, and *F* and *SI Appendix*, Fig. S3*G*). However, at 4 wk of age, significantly fewer growth plate chondrocytes were EdU^+^ in *Prx1^Cre^;Itga11^fl/fl^* as compared to control mice (Fig. 2*E* and *SI Appendix*, Fig. S3*G*). Cell death appeared to be rare in growth plate chondrocytes at all stages in *Prx1^Cre^;Itga11^fl/fl^* and control mice as we rarely observed TUNEL^+^ cells (*SI Appendix*, Fig. S4*E*). The number of Aggrecan^+^ chondrocytes per millimeter of growth plate was similar in *Prx1^Cre^;Itga11^fl/fl^* and control mice at all stages (Fig. 2 *G–J*). This suggested that Osteolectin/Integrin α11 signaling promotes bone elongation by increasing growth plate chondrocyte proliferation around 4 wk of age.

### Integrin α11 Acts Cell Autonomously in Chondrocytes to Promote Proliferation.

**Fig. 3.**
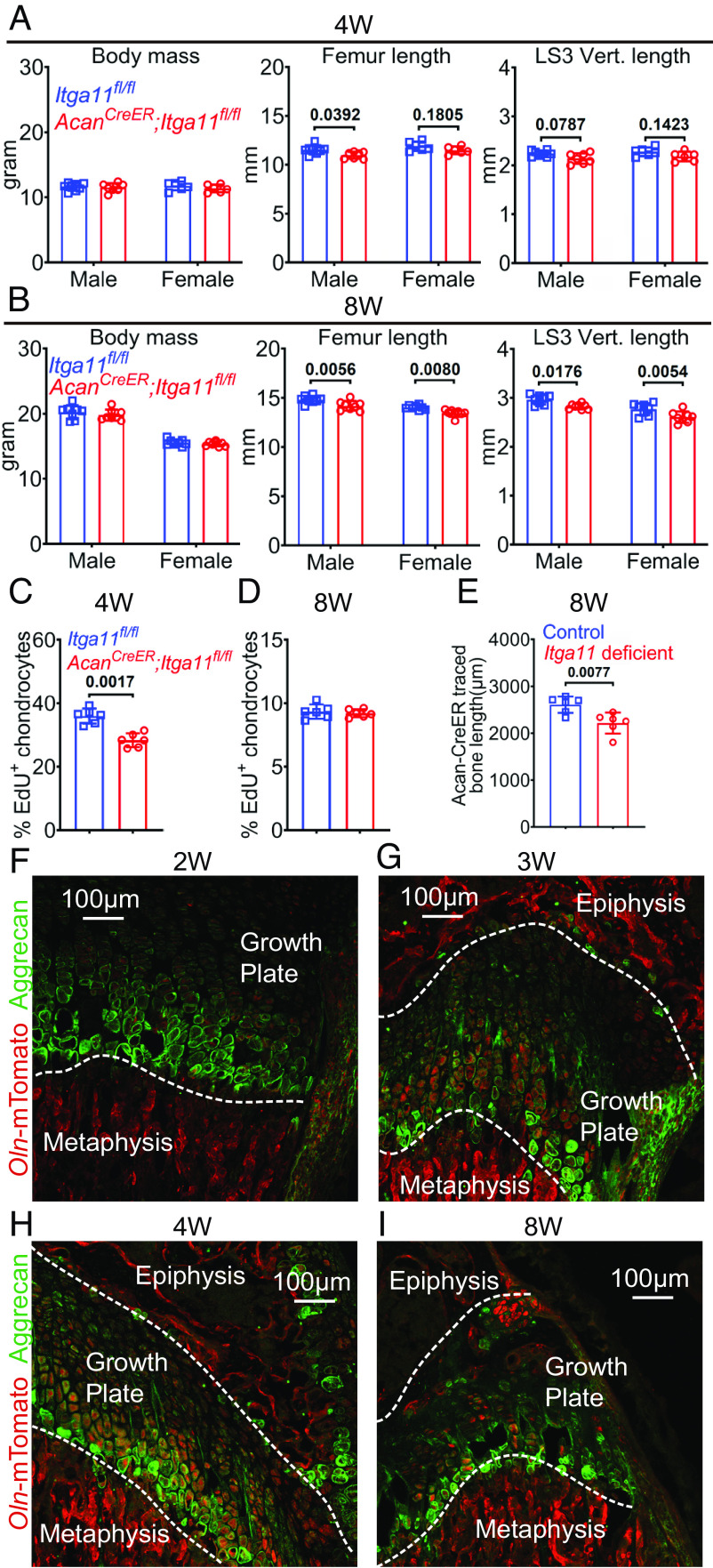
Integrin α11 cell-autonomously promoted the proliferation of growth plate chondrocytes. (*A* and *B*) *Acan^CreER^;Itga11^fl/fl^* and sex-matched littermate control (*Itga11^fl/fl^*) mice were treated with tamoxifen at 2 wk of age, then body mass, femur length, and LS3 vertebra length were measured at 4 wk (*A*) and 8 wk (*B*) of age. Each square/circle represents a different mouse (Six to eight mice per sex per age per genotype in four or five independent experiments per age). (*C* and *D*) The percentages of Aggrecan^+^ growth plate chondrocytes that were EdU^+^ at 4 wk (*C*) and 8 wk (*D*) of age (Three mice per sex per genotype per age in four or three independent experiments per age). (*E*) The length of cortical bone that arose from chondrocytes since tamoxifen treatment (Three mice per sex per genotype in three independent experiments). (*F*–*I*) Representative images of distal femur growth plate from *Osteolectin^mTomato/+^* mice at 2 (*F*), 3 (*G*), 4 (*H*), or 8 (*I*) wk of age. Dotted white lines indicate the boundary between the growth plate and metaphysis or epiphysis. All statistical tests were two sided. All data represent mean ± SD. Statistical significance was assessed using two-way ANOVAs followed by Sidak’s multiple comparison tests (*A* and *B*), Student’s *t* tests followed by Holm–Sidak's multiple comparison tests (*C* and *D*), or Student’s *t* tests (*E*).

To test whether Integrin α11 acted cell autonomously or noncell autonomously to promote the proliferation of growth plate chondrocytes, we conditionally deleted *Itga11* using *Aggrecan^CreER^* (*Acan^CreER^*), which recombines in chondrocytes (29). We initiated recombination at 2 wk of age by injecting *Acan^CreER^;Itga11^fl/fl^* mice and sex-matched *Itga11^fl/fl^* littermate controls with tamoxifen and then analyzed bone parameters at 4 wk and 8 wk of age. At both ages, *Acan^CreER^;Itga11^fl/fl^* mice and sex-matched *Itga11^fl/fl^* littermate controls did not differ in body mass (Fig. 3 *A* and *B*), femur cortical bone parameters (*SI Appendix*, Fig. S5 *A* and *B*), or femur or vertebra trabecular bone parameters (*SI Appendix*, Fig. S5 *C*–*F*). At 4 wk of age, there was a significant reduction or a trend toward shorter femurs and LS3 vertebrae in *Acan^CreER^;Itga11^fl/fl^* mice (Fig. 3*A*). At 8 wk of age, the *Acan^CreER^;Itga11^fl/fl^* mice had femurs and LS3 vertebrae that were significantly (4 to 6%) shorter than those in littermate controls (Fig. 3*B*). Integrin α11 thus acted within chondrocytes to promote bone elongation.

To test whether Integrin α11 promoted bone elongation by promoting the proliferation of growth plate chondrocytes, we analyzed *Acan^CreER^;Itga11^fl/fl^* mice and sex-matched *Itga11^fl/fl^* littermate controls at 4 and 8 wk of age. At both ages, the number of Aggrecan^+^ chondrocytes per millimeter of growth plate was similar in *Acan^CreER^;Itga11^fl/fl^* and sex-matched littermate control mice (*SI Appendix*, Fig. S5 *G–I*). The percentage of Aggrecan^+^ chondrocytes in the growth plate that incorporated a 2-d pulse of EdU was similar in *Acan^CreER^;Itga11^fl/fl^* and littermate controls at 8 wk of age (*SI Appendix*, Fig. S5*G* and Fig. 3*D*) but was lower in *Acan^CreER^;Itga11^fl/fl^* mice at 4 wk of age (*SI Appendix*, Fig. S5*G* and Fig. 3*C*). We did not detect any TUNEL^+^ chondrocytes in the growth plates of either *Acan^CreER^;Itga11^fl/fl^* or control mice at 4 or 8 wk of age (*SI Appendix*, Fig. S5*J*). Integrin α11 thus acted within growth plate chondrocytes to promote their proliferation, transiently increasing bone growth in juvenile mice.

Although deficiency for either *Osteolectin* or *Itga11* decreased the proliferation of growth plate chondrocytes at 4 wk of age (Figs. 1*E* and 3*C*), neither affected the number of Aggrecan^+^ chondrocytes per mm of growth plate (Fig. 1*I* and *SI Appendix*, Fig. S5*H*). Since growth plate chondrocyte proliferation lengthens bones as a result of the differentiation of chondrocytes into bone (26, 27), we wondered whether decreased chondrocyte proliferation was associated with reduced cortical bone generation. To test this, we generated *Acan^CreER^; Rosa26^loxp-tdTomato/+^* and *Acan^CreER^; Rosa26^loxp-tdTomato/+^; Itga11^fl/fl^* mice to trace the bone formed by chondrocytes in the presence and absence of Integrin α11. We treated the mice with tamoxifen at 2 wk of age and analyzed Tomato expression at 8 wk of age (*SI Appendix*, Fig. S6*A*). To quantitate the rate at which chondrocytes generated cortical bone, we measured the length of cortical bone in sections that contained Tomato^+^ bone cells, starting at the growth plate (white lines in *SI Appendix*, Fig. S6*A*). *Acan^CreER^; Rosa26^loxp-tdTomato/+^; Itga11^fl/fl^* mice had a significantly shorter length of Tomato^+^ cortical bone as compared to *Acan^CreER^; Rosa26^loxp-tdTomato/+^* controls (Fig. 3*E*). This suggested that Integrin α11 signaling promoted bone elongation by increasing growth plate chondrocyte proliferation and cortical bone generation.

We also tested whether *Acan^CreER^* recombined in perichondrial cells. Perichondrial cells contribute to bone formation in the diaphysis during fetal and early postnatal development (30). To test whether *Acan^CreER^* recombined in perichondrial cells, we treated 2-wk-old *Acan^CreER^; Rosa26^loxp-tdTomato/+^* mice with tamoxifen and then assessed Tomato expression 2 d later. Acan-CreER recombined in all or nearly all growth plate chondrocytes but only in a very small number of Periostin^+^ perichondrial cells (31) (*SI Appendix*, Fig. S6*B*). Within the diaphysis, where perichondrial cells give rise to cortical bone, we observed no recombination by  *Acan^CreER^* (*SI Appendix*, Fig. S6*C*). The recombination of Acan-CreER in perichondrial cells, thus, cannot explain the bone growth phenotype we observed.

To test whether there is a source of Osteolectin near the growth plate, we examined *Osteolectin^mTomato/+^* reporter mice (16). At 3 and 4 wk of age, we observed Tomato expression by bone lineage cells in the metaphysis and epiphysis, adjacent to the growth plate, as well as by chondrocytes within the growth plate (Fig. 3 *G* and *H*). We observed much less Tomato expression within the growth plate at 2 wk of age (Fig. 3*F*), and Tomato expression within the growth plate appeared to decline between 4 and 8 wk of age (Fig. 3*I*). *Osteolectin* expression thus increased within the growth plate at 3 to 4 wk of age, when it increased the proliferation of growth plate chondrocytes. This transient increase in Osteolectin expression within the growth plate at 3 to 4 wk of age may be part of the reason why bone elongation occurs more rapidly in juvenile as compared to adult mice.

### Recombinant Osteolectin Promotes Bone Elongation.

**Fig. 4.**
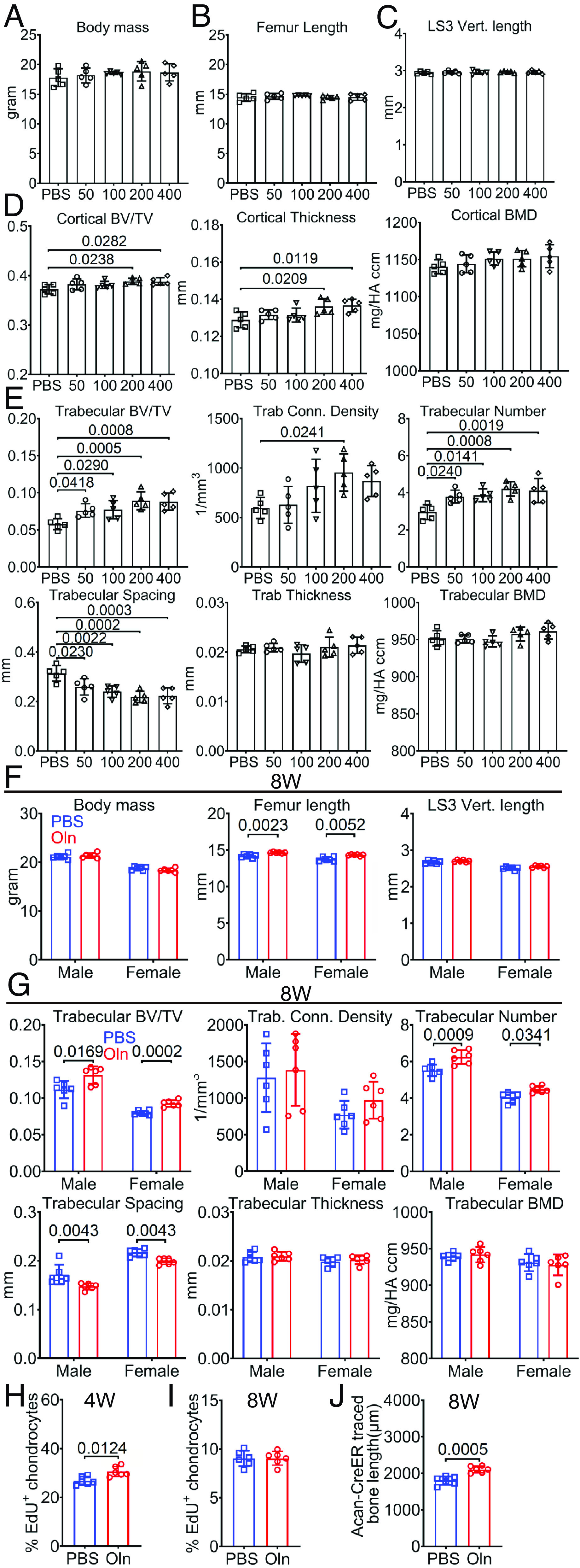
Daily injections of recombinant Osteolectin increased bone formation in mice. (*A*–*E*) Eight-week-old male wild-type mice were administered daily injections of PBS or recombinant Osteolectin (Oln) at 50 µg/kg/d (50), 100 µg/kg/d (100), 200 µg/kg/d (200), or 400 µg/kg/d (400) for 4 wk. Oln injections did not affect body mass (*A*), femur length (*B*), LS3 vertebra length (*C*), but 200 or 400 µg/kg/d significantly increased cortical bone volume/total volume and cortical thickness in the mid-femur diaphysis (*D*). Oln also significantly increased trabecular bone volume/total volume, connectivity density, number, and reduced trabecular spacing in the distal femur metaphysis (*E*) (Five mice per group in three independent experiments). (*F* and *G*) Wild-type mice were administered daily injections of PBS or recombinant Osteolectin (Oln) from 2 to 8 wk of age, then body mass, femur length, and LS3 vertebra length were measured at 8 wk of age (*F*), and Oln injection significantly increased trabecular bone volume/total volume and trabecular number and reducing trabecular spacing (*G*) (Six mice per sex per group in three independent experiments). (*H* and *I*) Percentages of Aggrecan^+^ growth plate chondrocytes that incorporated a 2-d pulse of EdU at 4 (*H*) or 8 (*I*) wk of age (Three mice per sex per age per group in three or four independent experiments per age). (*J*) *Acan^CreER^;Rosa26^loxp-tdTomato/+^* mice were administered daily injections of PBS or Oln from 2 to 8 wk of age, then the length of cortical bone that arose from chondrocytes since tamoxifen treatment was measured as in *SI Appendix*, Fig. S6*A* (Three mice per sex per group in three independent experiments). All statistical tests were two sided. All data represent mean ± SD. Statistical significance was assessed using one-way ANOVAs followed by Dunnett’s multiple comparison tests (*A*–*E*), Welch’s *t* test followed by Holm–Sidak’s multiple comparison test (femur length in *F*), Student’s *t* tests followed by Holm–Sidak’s multiple comparison tests (trabecular BV/TV and trabecular connectivity density in *G*–*I*), Mann–Whitney tests followed by Holm–Sidak’s multiple comparison tests (trabecular spacing in *G*), and two-way ANOVAs followed by Sidak’s multiple comparison tests (body mass and LS3 vertebra length in *F*, and other panels in *G*), or Student’s *t* tests (*J*).

With the goal of testing whether increased Osteolectin levels are sufficient to promote bone elongation in vivo, we first identified an effective dose of recombinant Osteolectin by injecting 8-wk-old male wild-type mice with 50 µg/kg/d, 100 µg/kg/d, 200 µg/kg/d, or 400 µg/kg/d Osteolectin for 1 mo. Control mice were injected daily with diluent (phosphate-buffered saline, PBS). None of these doses had any effect on body mass, femur length, or LS3 vertebra length (Fig. 4 *A*–*C*), consistent with the fact that bone elongation is largely complete by 8 wk of age (32). The lower doses (50 µg/kg/d and 100 µg/kg/d) did not affect cortical bone parameters, but the higher doses (200 µg/kg/d and 400 µg/kg/d) significantly increased femur cortical bone volume and thickness (Fig. 4*D*). All doses significantly increased trabecular bone volume and number (Fig. 4*E*). The data thus indicated that 200 µg/kg/d of recombinant Osteolectin increased bone formation in vivo.

To test the effect of Osteolectin on bone elongation, we injected 2- to 8-wk-old male and female wild-type mice with 200 µg/kg/d of recombinant Osteolectin or PBS. At 8 wk of age, the Osteolectin-treated and sex-matched littermate control mice did not differ in body mass or LS3 vertebra length (Fig. 4*F*). However, the Osteolectin-treated mice had femurs that were 3 to 4% longer than those of controls (Fig. 4*F*). The femurs from the Osteolectin-treated mice also had significantly increased trabecular bone volume and number and reduced trabecular spacing as compared to control femurs (Fig. 4*G*). The percentage of Aggrecan^+^ chondrocytes in the growth plate that incorporated EdU was higher in the Osteolectin-treated as compared to control mice at 4 wk of age (Fig. 4*H*) but similar in the Osteolectin-treated and control mice at 8 wk of age (Fig. 4*I*). Osteolectin treatment also significantly increased the length of cortical bone formed by chondrocytes in *Acan^CreER^; Rosa26^loxp-tdTomato/+^* mice (Fig. 4*J*; akin to the experiment in Fig. 3*E*). Recombinant Osteolectin is thus sufficient to promote bone elongation in juvenile mice and appeared to do so by increasing the proliferation of growth plate chondrocytes.

### Osteolectin/Integrin α11 Activates the Wnt Pathway in Chondrocytes.

**Fig. 5.**
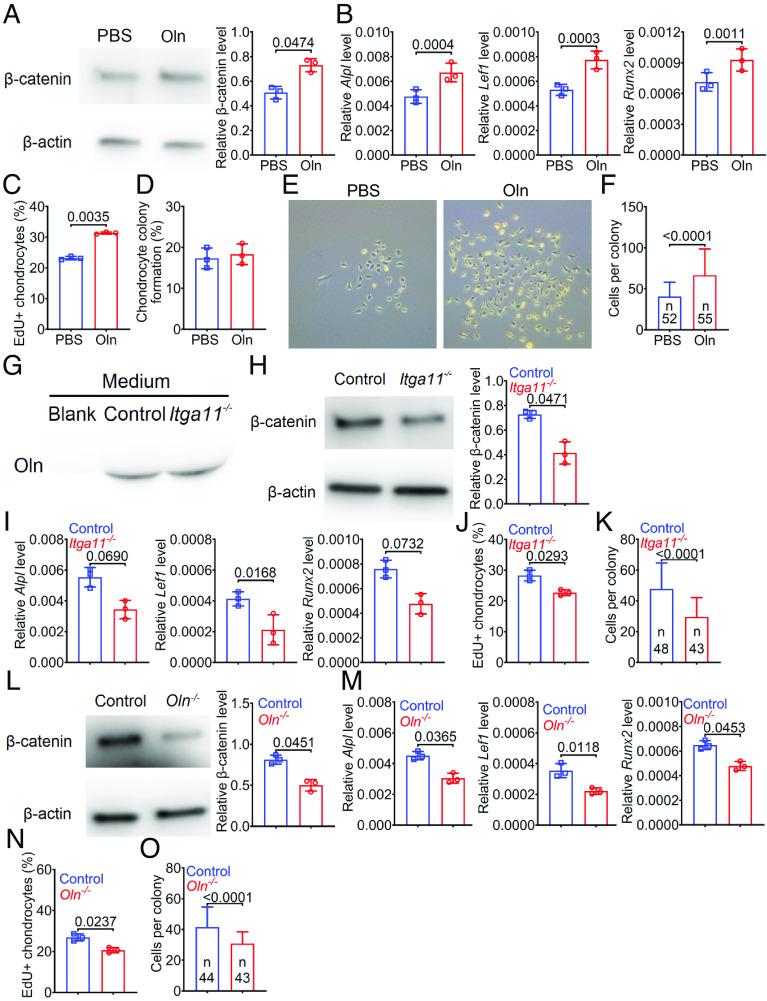
Osteolectin/Integrin α11 signaling promoted Wnt pathway activation and proliferation in chondrocytes. (*A*–*F*) Growth plate chondrocytes from 4-wk-old male wild-type mice were cultured with PBS or Oln for 2 d. β-catenin levels were quantified by western blot (*A*); *Alpl, Lef1,* and *Runx2* transcripts were quantified by qRT-PCR (*B*); and EdU incorporation was quantitated by flow cytometry (*C*). Chondrocytes from these cultures were subcloned into secondary cultures at clonal density (100 cells per well in six-well dishes), and the percentage of cells that formed colonies (*D*) as well as colony size was assessed (*E* and *F*) in the presence and absence of Oln (Three mice per group in panels *A*–*E*, 52 and 55 colonies per group in panel *F*, all in three independent experiments). (*G*–*K*) Growth plate chondrocytes from 4-wk-old male *Acan^CreER^;Itga11^fl/fl^* and littermate *Itga11^fl/fl^* control mice were cultured with 4-hydroxytamoxifen for 2 d to delete Integrin α11. Osteolectin expression by these cells was confirmed by western blotting of the culture medium (*G*). In the cells, β-catenin protein levels (*H*); *Alpl, Lef1,* and *Runx2* transcript levels (*I*); and EdU incorporation were quantified (*J*). Chondrocytes from these cultures were subcloned into secondary cultures at clonal density and the number of cells per colony was counted (*K*) (Three mice per genotype in panels *G*–*K*, 48 and 43 colonies per genotype in panel *K*, all in three independent experiments). (*L*–*O*) Growth plate chondrocytes from 4-wk-old male *Oln^−/−^* and littermate control mice were cultured for 2 d and then β-catenin protein levels (*L*); *Alpl, Lef1,* and *Runx2* transcript levels (*M*); and EdU incorporation were quantified (*N*). Chondrocytes from these cultures were subcloned into secondary cultures at clonal density and the number of cells per colony was counted (*O*) (Three mice per genotype in panels *L*–*N*, 44 and 43 colonies per genotype in panel *O*, all in three independent experiments). All statistical tests were two sided. All data represent mean ± SD. Statistical significance was assessed using paired *t* tests (*A*, *C*, *D*, *H*, *J*, *L*, and *N*), paired sample two-way ANOVAs followed by Sidak’s multiple comparisons test (*B*, *I*, and *M*), or Student’s *t* tests (*F*, *K*, and *O*).

Wnt pathway activation promotes the proliferation and osteogenic differentiation of chondrocytes (33). To test whether Osteolectin/Integrin α11 signaling increases Wnt pathway activation in chondrocytes, we cultured growth plate chondrocytes from 4-wk-old wild-type mice. The Osteolectin-treated cells had increased levels of total β-catenin (Fig. 5*A*) and active β-catenin (unphosphorylated at GSK-3-dependent sites including Ser33, Ser37, and Thr41) (*SI Appendix*, Fig. S7*A*), and Wnt target genes, including *Alpl* (34), *Lef1* (35), and *Runx2* (36) (Fig. 5*B*), as compared to control chondrocytes. The Osteolectin-treated chondrocytes also exhibited increased EdU incorporation as compared to control cells (Fig. 5*C*). When the chondrocytes were subcloned into secondary cultures at clonal density, Osteolectin did not affect the percentage of cells that formed colonies (Fig. 5*D*) but increased the size of colonies (Fig. 5 *E* and *F*). Osteolectin did promote osteogenic differentiation by chondrocytes cultured in osteogenic differentiation medium (*SI Appendix*, Fig. S7*B*). Osteolectin thus promotes Wnt pathway activation, proliferation, and osteogenic differentiation in chondrocytes.

Bone marrow stromal cells secrete Osteolectin into the culture medium and it promotes their differentiation into osteoblasts (19). To test whether chondrocytes secrete Osteolectin that promotes their proliferation, growth plate chondrocytes from 4-wk-old *Acan^CreER^;Itga11^fl/fl^* and sex-matched *Itga11^fl/fl^* littermate control mice were cultured with 4-hydroxytamoxifen for 2 d to delete *Itga11*. Western blotting showed the presence of Osteolectin in the culture medium (Fig. 5*G*). The *Itga11*-deficient cultures had significantly lower levels of total β-catenin (Fig. 5*H*); β-catenin unphosphorylated at GSK-3-dependent sites (*SI Appendix*, Fig. S7*C*); *Alpl*, *Lef1,* and *Runx2* expression (Fig. 5*I*); EdU incorporation (Fig. 5*J*); cells per colony (Fig. 5*K*); and osteogenic differentiation (*SI Appendix*, Fig. S7*D*) as compared to control cultures. Chondrocytes thus secrete Osteolectin and Osteolectin/Integrin α11 signaling in chondrocytes promotes Wnt pathway activation, proliferation, and osteogenic differentiation.

Consistent with this, *Osteolectin*-deficient growth plate chondrocytes had significantly lower levels of total β-catenin (Fig. 5*L*); unphosphorylated β-catenin (*SI Appendix*, Fig. S7*E*); *Alpl*, *Lef1,* and *Runx2* expression (Fig. 5*M*); EdU incorporation (Fig. 5*N*); cells per colony (Fig. 5*O*); and osteogenic differentiation (*SI Appendix*, Fig. S7*F*) compared to control chondrocytes.

To test whether reduced β-catenin (encoded by *Ctnnb1*) levels phenocopy *Osteolectin* or *Itga11* deficiency, we treated *Acan^CreER^;Ctnnb1^fl/+^* and sex-matched *Ctnnb1^fl/+^* littermate control mice with tamoxifen at 2 wk of age and then analyzed them at 4 and 8 wk of age. At both ages, the *Acan^CreER^;Ctnnb1^fl/+^* mice had femurs and LS3 vertebrae that were significantly (5 to 6%) shorter than those in littermate controls (*SI Appendix*, Fig. S7 *G* and *H*). *Acan^CreER^;Ctnnb1^fl/+^* mice and littermate controls did not significantly differ in body mass, femur cortical bone volume, or femur or vertebral trabecular bone volume (*SI Appendix*, Fig. S7 *G* and *H*). The percentage of Aggrecan^+^ chondrocytes in the growth plate that incorporated a 2-d pulse of EdU was similar in *Acan^CreER^;Ctnnb1^fl/+^* and littermate controls at 8 wk of age (*SI Appendix*, Fig. S7*J*) but was lower in *Acan^CreER^;Ctnnb1^fl/+^* mice at 4 wk of age (*SI Appendix*, Fig. S7*I*). A quantitative reduction in b-catenin levels within growth plate chondrocytes, thus, transiently reduced chondrocyte proliferation and bone elongation, just as observed in *Osteolectin* and *Itga11* mutant mice. Osteolectin/Integrin α11 signaling in growth plate chondrocytes thus appears to promote the expression of Wnt target genes by increasing b-catenin activity.

### The rs182722517 Variant Reduces Osteolectin Expression by hBMSCs.

**Fig. 6.**
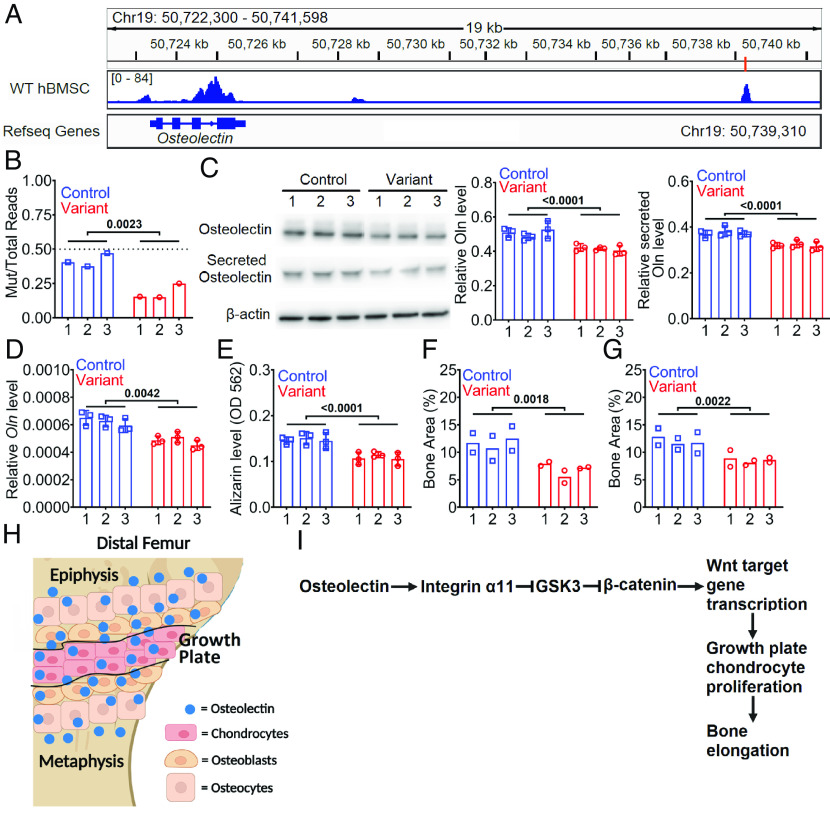
The rs182722517 variant reduced Osteolectin expression and osteogenic differentiation by human bone marrow stromal cells. (*A*) ATAC sequencing of primary human bone marrow stromal cells (hBMSCs) showing a peak of accessible chromatin at chr19:50739310, where the rs182722517 variant is observed (these cells contained the common allele). (*B*) ATAC sequencing in heterozygous rs182722517 variant–containing or control hBMSCs determined the ratio of reads from the CRISPR-edited allele versus the endogenous allele in each clone. This revealed a significantly lower fraction of reads from the rs182722517 variant–containing allele as compared to the CRISPR-edited control allele, suggesting that the rs182722517 variant reduces chromatin accessibility (Three independently targeted clones per genotype). (*C* and *D*) Osteolectin levels were assessed in cell extracts and in culture medium from cultures of homozygous rs182722517 variant–containing or control hBMSCs by western blot (*C*) or qRT-PCR (*D*) (Three replicate cultures per clone in three independent experiments). (*E*−*G*) Osteogenic differentiation of homozygous rs182722517–containing or control hBMSCs based on Alizarin Red staining in culture (*E*; Three replicates per clone from three independent experiments), or the area occupied by bone in sections through ossicles that grew in vivo. The sections were stained with hematoxylin and eosin (*F*) or trichome (*G*) to identify bone (Two replicates per clone from two independent experiments). (*H*) Osteolectin produced in, and around, the growth plate activates Integrin α11 signaling in growth plate chondrocytes, promoting chondrocyte proliferation and bone elongation (image generated using BioRender). (*I*) Schematic summarizing mechanism. All statistical tests were two sided. All data represent mean ± SD. Statistical significance was assessed using a Student’s *t* test (*B*) and nested *t* tests (*C*−*G*).

Separate genome-wide association studies in humans identified a single-nucleotide variant (rs182722517) that is associated with reduced height (22–24) and plasma Osteolectin levels (21). Rs182722517 is located at chr19:50739310 [GRCh38 (37)], 16 kb downstream of *Osteolectin*. The major allele at this locus is G, the rs182722517 variant has an A, and the rs182722517 variant frequency is 0.0032 (38). Rs182722517 is located within a candidate cis-regulatory element, ranging from chr19:50739241-50739456, that is annotated by ENCODE (Expanded Encyclopedias of DNA Elements) (39, 40). To test whether this represents a possible cis-regulatory element in hBMSCs, we performed ATAC sequencing on primary human bone marrow stromal cells (hBMSCs) bearing the major allele and detected a strong peak of accessible chromatin in the region surrounding the rs182722517 locus (Fig. 6*A*).

This intergenic sequence differs in the mouse genome. To test whether the rs182722517 variant alters Osteolectin expression, we introduced this variant into wild-type hBMSCs using CRISPR/Cas9 editing (*SI Appendix*, Fig. S8*A* for the editing strategy). Prior to CRISPR editing, we confirmed that the hBMSCs carried the major allele at the chr19:50739310 locus (*SI Appendix*, Fig. S8*B*). To increase recombination efficiency, we introduced a C>G mutation in a protospacer adjacent motif (PAM, chr19:50739290) that prevented recutting of the already-recombined single-stranded oligodeoxynucleotide (ssODN) sequence. We isolated three independently targeted hBMSC clones with homozygous G>A mutations at the chr19:50739310 locus and PAM site mutations, three independently targeted hBMSC clones with heterozygous G>A mutations at the chr19:50739310 locus and PAM site mutations, three control clones with homozygous PAM mutations, and three control clones with heterozygous PAM mutations (*SI Appendix*, Fig. S8*B*).

To test whether the rs182722517 variant affects chromatin accessibility in hBMSCs, we performed ATAC sequencing on heterozygous variant and control hBMSCs. The PAM mutation did not appear to affect chromatin accessibility as there were approximately equal numbers of reads from the PAM mutation–containing allele and the wild-type allele in control cells that were heterozygous for the PAM mutation (Fig. 6*B*). Conversely, there were significantly fewer reads of the rs182722517 variant–containing allele than those of the wild-type allele in cells heterozygous for the rs182722517 variant (Fig. 6*B*). This suggests that the rs182722517 variant reduced chromatin accessibility in hBMSCs.

To test whether the rs182722517 variant affects Osteolectin expression, we compared Osteolectin production by variant and control hBMSCs in culture. Clones that were homozygous for the rs182722517 variant had lower levels of Osteolectin in cell extracts and lower levels of Osteolectin secreted into the culture medium as compared to control clones (Fig. 6*C*). *Osteolectin* mRNA levels were also lower in the variant clones (Fig. 6*D*). The rs182722517 variant thus reduced Osteolectin expression by hBMSCs.

We next tested the effect of the rs182722517 variant on osteogenesis by hBMSCs. As noted above, Osteolectin expression by hBMSCs promotes osteogenic differentiation in culture (19). After 21 d in osteogenic culture medium, homozygous variant clones exhibited significantly reduced Alizarin red levels as compared to control clones (Fig. 6*E* and *SI Appendix*, Fig. S8*C*). To test the effect of the rs182722517 variant on osteogenesis in vivo, we mixed homozygous variant or control cells with hydroxyapatite/tricalcium phosphate particles and fibrin gel, then transplanted the suspensions subcutaneously into 3- to 4-mo-old NOD.CB17-*Prkdc^scid^* *Il2rg^tm1Wjl^*/SzJ (NSG) mice and allowed them to form bony ossicles for 6 wk. The variant clones exhibited significantly reduced bone formation as compared to the control clones (Fig. 6 *F* and *G* and *SI Appendix*, Fig. S8 *D* and *E*). The rs182722517 variant thus reduced osteogenic differentiation by hBMSCs in vitro and in vivo.

## Discussion

Osteolectin/Integrin α11 signaling promotes the maintenance of adult bone mass by promoting the differentiation of LepR^+^ skeletal stem cells into osteoblasts (18, 19). Consistent with this, PTH increases plasma Osteolectin levels in people who exhibit an osteogenic response to PTH (20). Moreover, *Osteolectin* deficiency reduces the osteogenic response to PTH in mice (20). This raised the possibility that Osteolectin mediates much of the effect of PTH on osteogenesis in humans; however, genetic evidence that Osteolectin regulates osteogenesis in humans was lacking. In this study, we found that Osteolectin/Integrin α11 signaling promotes longitudinal bone growth in juvenile mice by increasing Wnt pathway activation and proliferation in growth plate chondrocytes. In humans, the rs182722517 variant is associated with reduced height (22–24) and plasma Osteolectin levels (21), suggesting that Osteolectin promotes bone elongation in humans as well. The rs182722517 variant is located in a candidate cis-regulatory element (Fig. 6*A*) that influences chromatin accessibility in hBMSCs (Fig. 6*B*). The rs182722517 variant reduced Osteolectin expression and osteogenic differentiation by hBMSCs (Fig. 6 *C*–*G*). Taken together, the data suggest that Osteolectin/Integrin α11 signaling promotes bone elongation in mice and humans, revealing a function for Osteolectin/Integrin α11 as well as genetic evidence that this function is conserved among mice and humans.

Osteolectin is expressed by LepR^+^ bone marrow stromal cells, osteoblasts, osteocytes, growth plate chondrocytes, and periosteal cells (Fig. 3 *F–I*). The bone elongation phenotype we report in this study is likely driven by Osteolectin that is expressed by chondrocytes within the growth plate as well as by osteoblasts/osteocytes in the metaphysis/epiphysis, adjacent to the growth plate (Fig. 3 *F*–*I*). The Osteolectin produced in, and around, the growth plate activates Integrin α11 signaling in growth plate chondrocytes (Fig. 6*H*). Integrin α11 signaling promotes β-catenin activation, increasing the transcription of Wnt target genes, the division of growth plate chondrocytes, and bone elongation (Fig. 6*I*). These effects appear to be driven by an increase in Osteolectin expression within the growth plate at around 3 wk after birth (Fig. 3 *F*–*I*), increasing growth plate chondrocyte proliferation and bone elongation starting before 4 wk of age and ending before 8 wk of age. A transient increase in Osteolectin/Integrin α11 signaling in growth plate chondrocytes thus appears to be part of the reason why bone elongation is more rapid in juvenile as compared to adult mice.

We previously reported that Osteolectin/Integrin α11 signaling in mouse LepR^+^ bone marrow stromal cell inhibits GSK3, promoting the accumulation of β-catenin and the transcription of Wnt target genes (19). The current study suggests that Osteolectin/Integrin α11 signaling promotes the proliferation and osteogenic differentiation of growth plate chondrocytes through a similar signaling mechanism (Fig. 5 and *SI Appendix*, Fig. S*7*). This is also consistent with studies by other groups who showed that increased Wnt pathway activation is necessary and sufficient to promote growth plate chondrocyte proliferation and bone formation (5, 36, 41).

A limitation is that human growth plate chondrocytes bearing the rs182722517 variant were not available to us. Consequently, we had to study the effect of the rs182722517 variant on Osteolectin expression and osteogenic differentiation in human bone marrow stromal cells.

Skeletal stem/progenitor cells (SSCs) that contribute to bone repair are present in the periosteum on the outside surface of bones (42–44) as well as inside the bone marrow (12, 45–47). We recently compared the functions of Gli1^+^ periosteal SSCs to LepR^+^ bone marrow SSCs and found that they make distinct contributions to the maintenance and repair of bones (47). LepR^+^ bone marrow SSCs are responsible for steady-state osteogenesis and the repair of drill injuries in bone, while Gli1^+^ periosteal SSCs are responsible for the repair of bicortical fractures as well as the regeneration of LepR^+^ bone marrow stromal cells at fracture sites. The observation that Osteolectin is expressed in periosteal cells (16) and that *Osteolectin* deficiency slows fracture repair (18) raises the possibility that Osteolectin may promote osteogenesis by periosteal cells. This will be an important issue to address in future studies, given that past studies have focused on the effect of Osteolectin on LepR^+^ bone marrow SSCs.

## Materials and Methods

### Mice.

All mouse experiments complied with all relevant ethical regulations and were performed according to protocols approved by the Institutional Animal Care and Use Committee at UT Southwestern Medical Center (UTSW; protocol 2017-101896). Human bone marrow stromal cells were transplanted into NOD.CB17-*Prkdc^scid^* *Il2rg^tm1Wjl^*/SzJ (NSG) mice in ossicle formation assays (see details below). All other mice were maintained on a C57BL/Ka background, including *Osteolectin^−/−^* (*Oln^−/−^*) mice (18), *Prx1^Cre^* mice (28), *Itga11^flox^* mice (19), *Aggrecan^CreER^(Acan^CreER^)* mice (29), *Ctnnb1^flox^* mice (48), *Rosa26*-*CAG*-*loxp*-*stop*-*loxp*-*tdTomato* (*Rosa^loxp-tdTomato^*) mice (49), and *Osteolectin^mTomato^* (*Oln^mT^*) mice (16). To induce Cre recombinase activity, *Acan^CreER^* mice were given three intraperitoneal injections of tamoxifen dissolved in corn oil at a dose of 100 mg/kg body mass/injection over 5 d. Mouse body mass was measured using a Scout SPX123 Precision Balance (Ohaus). All mice were housed in AAALAC-accredited, specific pathogen-free animal care facilities at UTSW. Primers used to genotype mice are shown in the methods section of *SI Appendix*.

In some experiments, recombinant mouse Osteolectin was administered to 2-mo-old wild-type male mice by daily subcutaneous injections for 1 mo, at a dose of 50 µg/kg/d, 100 µg/kg/d, 200 µg/kg/d, or 400 µg/kg/d. One hundred microliters of PBS was injected as vehicle control. Daily Osteolectin injections were also administered subcutaneously to wild-type mice from 2 to 8 wk of age at a dose of 200 µg/kg/d. Osteolectin was purified as previously described (18, 19) by affinity purification after secretion into the culture medium by Osteolectin overexpressing H293 cells.

### Generation and Culture of Genetically Engineered Human Bone Marrow Stromal Cells.

Primary human bone marrow stromal cells (hBMSCs) were acquired from Lonza (PT-2501). The cells were cultured in DMEM (Gibco) with 15% fetal bovine serum (FBS) (Sigma, F0926) at 37 °C in a 5% CO_2_ incubator, and the medium was refreshed every 2 d. The cells were confirmed to carry the major allele G at the rs182722517 locus. To generate hBMSCs carrying the minor allele A at the variant site, two gRNAs were designed to target a site 22 base pairs upstream of the variant site. Two ssODNs were designed for recombination. The control ssODN carried a C>G mutation at the PAM to prevent recutting after the ssODN had recombined into the genome, thus increasing editing efficiency. The variant ssODN carried this PAM mutation as well as a G>A mutation at the variant site. For transfection, cells were dissociated using TrypLE™ Express Enzyme (Gibco). Approximately 3 × 10^5z^ to 5 × 10^5^ cells were transfected with Cas9 ribonucleoprotein complex (200 μM crRNA-tracrRNA complex + 15 μg Cas9) and ssODN (12 μg) following P1 Primary Cell 4D-Nucleofector™ X Kit Protocol FF-104.

The sequences of gRNAs and ssODNs are listed below: gRNA1 was 5′-CAC TCA TGT GTC TCT AGT TA_TGG; gRNA2 was 5′-ACT CAT GTG TCT CTA GTT AT_GGG; control ssODN was 5′-TCA CGT TCT CCT CCA CCA TAG CAC AGA GCG TCT AAG GGT GCC ACC CTC TC**G** CAT AAC TAG AGA CAC ATG AGT GAC AGC AGC AAT GAG CTG TCC CAT CTG CTA GTC GTC GAC ACA GAA GAG C (PAM mutation site is in bold); and rs182722517 variant ssODN was 5′- TCA CGT TCT CCT CCA CCA TAG CAC AGA GCG TCT AAG GGT GCC ACC CTC TC**G** CAT AAC TAG AGA CAC ATG A**A**T GAC AGC AGC AAT GAG CTG TCC CAT CTG CTA GTC GTC GAC ACA GAA GAG C (the PAM and the variant mutations are in bold).

Transfected cells were cultured at high density for 5 d and then single cells were sorted into 96-well plates and grown for 2 to 3 wk. To genotype the resulting colonies, genomic DNA was extracted using Quick-DNA 96 Kits (Zymo Research) and amplified by PCR using the following primers: forward 5′- CTT CAC GTA TT CAT TCA CGC A and reverse 5′- AAA GTG GAG TCG GTA GGT CA. The PCR products were then subjected to Sanger DNA sequencing using the sequencing primer: 5′- GTC AGA CGT TCA GTT AAG AAC TGC.

### Mouse Growth Plate Chondrocyte Cultures.

Mouse growth plate chondrocytes were dissected according to published protocols (50, 51), with modifications described below. The cartilage cap of the femoral heads was removed from 4-wk-old mice using a blunt forceps. The cartilage caps were placed in 50 mL conical tubes containing PBS with penicillin and streptomycin (1:10 dilution of HyClone Penicillin-Streptomycin Solution from Cytiva). The cartilage caps were washed twice with PBS and then incubated in 5 mL pronase solution (Sigma Aldrich; 1 mg/mL pronase in DMEM with 5% FBS and 0.5% penicillin and streptomycin, sterilized by 0.22 µm filtration) in a 60-mm culture dish for 1 h at 37 °C in a 5% CO_2_ incubator. Pronase solution was removed, and the cartilage caps were washed once with PBS. They were then digested with liberase solution (Sigma Aldrich; 1 mg/mL liberase in DMEM with 1% FBS, sterilized by 0.22 µm filtration) for 6 h at 37 °C in a 5% CO_2_ incubator. Chondrocytes were liberated by pipetting the remaining cartilage fragments up and down a dozen times and filtering through a 40-µm cell strainer. The cells were then washed twice with PBS and pelleted by centrifuging at 500× g for 5 min. These cells were then cultured in complete medium (DMEM with 10% FBS plus 1% penicillin and streptomycin) overnight at 37 °C in a 5% CO_2_ incubator. Floating cells were removed, and viable chondrocytes were allowed to attach to the plastic surface of cell culture dish.

Chondrocytes from 4-wk-old *Acan^CreER^;Itga11^fl/fl^* and *Itga11^fl/fl^* control mice were cultured with 200 nM 4-hydroxytamoxifen (Sigma Aldrich) in complete medium at 37 °C in a 5% CO_2_ incubator for 2 d, then washed with complete medium to remove 4-hydroxytamoxifen. The chondrocytes were cultured with 40 ng/mL recombinant Osteolectin (obtained as described below) in complete medium at 37 °C in a 5% CO_2_ incubator for 2 d. To quantitate proliferation, the chondrocytes were cultured with 10 µM EdU in complete medium for 24 h and then analyzed by flow cytometry. Alternatively, 2 d after 4-hydroxytamoxifen treatment, the chondrocytes were subcloned into secondary cultures at clonal density (100 cells/well in six-well culture dishes) in complete medium for 5 d, and then the colonies were imaged using a Leica DMi1 Inverted Microscope to count colony numbers and cells per colony.

Chondrocytes were cultured at 10,000 cells per well in osteogenic differentiation medium (StemPro Osteogenesis Differentiation kit, Gibco) in 48-well tissue culture plates for 3 wk to induce osteogenic differentiation. These cultures were then stained with Alizarin Red S (EMD Millipore). To quantitate Alizarin red staining, the stained cells were rinsed with PBS and extracted with 10% (w/v) cetylpyridinium chloride in 10 mM sodium phosphate (pH 7.0) for 10 min at room temperature. Alizarin red in the extract was quantitated by optical density measurement at 562 nm.

### MicroCT Analysis.

MicroCT analysis was performed using the settings described previously (18, 19). Mouse femurs were dissected, fixed overnight in 4% paraformaldehyde (Thermo Fisher Scientific), and stored in 70% ethanol at 4 °C (52). The femurs and lumbar spine vertebrae were scanned at an isotropic voxel size of 3.5 μm and 7 μm, respectively, with a peak tube voltage of 55 kV and current of 0.145 mA (μCT 35; Scanco). A three-dimensional Gaussian filter (s = 0.8) with a limited, finite filter support of one was used to suppress noise in the images, and a threshold of 330 to 1,000 was used to segment mineralized bone from air and soft tissues. Trabecular bone parameters were measured in the femur distal metaphysis. The region was selected from below the distal growth plate where the epiphyseal cap structure completely disappeared and continued for 100 slices toward the proximal end of the femur. Contours were drawn manually a few voxels away from the endocortical surface to define trabecular bone in the metaphysis. Cortical bone parameters were measured by analyzing 100 slices in mid-diaphysis femurs. Vertebral trabecular bone parameters were measured by analyzing the vertebral body of the third lumbar spine vertebra (LS3). The region started from the top where the vertebral body fully connects to the transverse process and continued 100 slices toward the bottom of the vertebra. For methods related to the immunostaining of bone sections, see methods in *SI Appendix*.

### Osteogenic Differentiation of Human Bone Marrow Stromal Cells.

To assess osteogenic differentiation, hBMSCs cells were transferred into 48-well plates at confluent cell density (10,000 cells/well). On the second day after plating, the culture medium was replaced with osteogenic differentiation medium (StemPro Osteogenesis Differentiation kit, Gibco). The medium was replaced with fresh medium every 3 d. The cells were cultured for 21 d and their osteogenic differentiation was analyzed by staining with Alizarin red S (Sigma Aldrich). The cells were washed with PBS and then fixed in 4% paraformaldehyde (Thermo Fisher Scientific) for 10 min. They were then washed with PBS twice and stained with Alizarin Red S for 15 min, followed by washing with PBS another three times. The cells stained with Alizarin Red S were imaged using a Leica DMi1 Inverted Microscope. To quantitate Alizarin red staining, the stained cells were rinsed with PBS and extracted with 10% (w/v) cetylpyridinium chloride in 10 mM sodium phosphate (pH 7.0) for 10 min at room temperature. Alizarin red in the extract was quantitated by optical density measurement at 562 nm.

### Ossicle Formation by Human Bone Marrow Stromal Cells.

Bone ossicle formation by hBMSCs in vivo was assessed as described previously (53). hBMSC clones were cultured in osteogenic differentiation medium (StemPro Osteogenesis Differentiation kit, Gibco) for 5 d. Then 2 × 10^6^ cells per clone were incubated with 40 mg hydroxyapatite/tricalcium phosphate particles (65%/35%, Zimmer Dental), rotating for 2 h at 37 °C. The cell/carrier slurry was centrifuged at 135× g for 5 min and embedded in a fibrin gel by adding 15 µL human fibrinogen (3.2 mg/mL in sterile PBS; EMD Millipore) with 15 µL human thrombin (25 U/mL in sterile 2% CaCl_2_ in PBS; EMD Millipore). The suspensions were left at room temperature for 10 min to clot and then transplanted subcutaneously into NSG mice. After 6 wk in vivo, the bony ossicles formed by these cells were harvested and analyzed by cryosectioning of formalin-fixed, paraffin-embedded specimens followed by hematoxylin and eosin (H&E) staining or trichrome staining. We analyzed three sections from each bony ossicle with a 200-μm distance between sections. The bone area in the sections was determined based on H&E or trichrome staining and morphology. The percentage of bone area in each section was imaged and quantified with Virtual Slide Scanner NanoZoomer 2.0HT (Hamamatsu) and analyzed with NDP.view2 software (Hamamatsu).

### ATAC Sequencing and Data Analysis.

ATAC sequencing was performed based on a published protocol (54) with modifications described below. Briefly, 2 × 10^4^ hBMSCs were washed twice in PBS and resuspended in 500 μL lysis buffer (10 mmol/L Tris-HCl, 10 mmol/L NaCl, 3 mmol/L MgCl2, 0.1% NP-40, pH 7.4). After cell lysis, the nuclei were pelleted by centrifugation at 500× g for 10 min at 4 °C. The nuclei were then resuspended in 50 μL tagmentation mix (Illumina) and incubated at 37 °C for 45 min. The tagmentation reaction was terminated by adding 10 μL 0.2% SDS and incubating at room temperature for 2 min and then at 55 °C for 7 min. TDE1 transposase–tagged DNA was purified using the QIAquick MinElute PCR Purification Kit (Qiagen) and amplified using KAPA HiFi Hotstart PCR Kit (KAPA) with Nextera DNA CD Indexes (Illumina). Libraries were quantitated using the double-stranded DNA High-Sensitivity Assay Kit (Invitrogen) on the Qubit fluorometer and the Agilent 2,200 TapeStation and were sequenced on an Illumina Nextseq500 using the 75 bp high-output sequencing kit.

Wild-type hBMSCs were sequenced (56.8 M reads) as 75 bp single-end reads using an Illumina NextSeq 500. Control and variant hBMSCs were sequenced (61.8 M ± 4.0 M reads per sample) as 75 bp paired-end reads using an Illumina NextSeq 500. The quality of raw reads was checked using FastQC 0.11.8. Raw reads were trimmed using TrimGalore 0.6.4 and mapped to the Ensembl GRCh38 mouse reference genome version 100 using Bowtie 2.4.1. Mapped reads were quality-filtered using SAMtools 1.12 to keep reads of MAPQ score > 10. In wild-type samples, 72% of raw reads with a >10 MAPQ score were processed by deepTools 3.5.1 to generate bigwig files, and IGV version 2.11.9 was used to browse them and generate panel Fig. 6*A*. In CRISPR-edited samples, 52.7 ± 1.7% of the raw reads with a >10 MAPQ score were processed by Bowtie2.4.1 to generate bam files, and IGV version 2.11.9 was used to browse them to quantify wild-type allele reads and mutant allele reads. In the six heterozygous control and variant clones, only the reads spanning both the PAM (Chr19:50739290) and the variant loci (Chr19:50739310) that had no mutations at both sites were counted as wild-type allele reads, the reads having only C>G mutations at the PAM were counted as control edited allele reads, and the reads having both C>G mutations at the PAM and G>A mutations at the rs182722517 variant locus were counted as variant allele reads. Total reads were quantitated as the sum of the wild-type reads and the edited allele reads in each clone, and the ratio of the mutant reads to total reads is presented in Fig. 6*B*. ATAC sequencing data have been submitted to the NCBI Sequence Read Archive, accession number: BioProject PRJNA887449.

For methods related to quantitative PCR and western blots, see the methods section of *SI Appendix*.

### Statistical Analysis.

In each type of mouse experiment, multiple mice were tested in multiple independent experiments performed on different days. Mice were allocated to experiments randomly and samples processed in an arbitrary order, but formal randomization techniques were not used. No formal blinding was applied when performing the experiments or analyzing the data. Sample sizes were not predetermined based on statistical power calculations but were based on our experience with these assays. No data were excluded.

Prior to analyzing the statistical significance of differences among genotypes and treatments, we tested whether data were normally distributed and whether variance was similar among groups. To test for normality, we performed the Shapiro–Wilk tests when 3 ≤ n < 20 or D’Agostino Omnibus tests when n ≥ 20. To test whether variability significantly differed among groups, we performed F-tests (for experiments with two groups) or Levene’s median tests (for experiments with more than two groups). When the data significantly deviated from normality or variability significantly differed among groups, we log2-transformed the data and tested again for normality and variability. If the transformed data no longer significantly deviated from normality and equal variability, we performed parametric tests on the transformed data. If log2 transformation was not possible or the transformed data still significantly deviated from normality or equal variability, we performed nonparametric tests on the nontransformed data.

When data or log2-transformed data were normal and equally variable, statistical analyses were performed using Student’s *t* tests/paired *t* tests (when there were two groups), nested *t* tests (when there were two groups and, in each group, subjects had multiple measurements), one-way ANOVAs (when there were more than two groups), and two-way ANOVAs/matched samples two-way ANOVAs (when there were two or more groups with multiple genotypes, treatments, or sexes). When the data or log2-transformed data were normal but unequally variable, statistical analyses were performed using Welch’s *t* tests (when there were two groups). When the data and log2-transformed data were abnormal or unequally variable, statistical analysis was performed using Mann–Whitney tests (when there were two groups). *P*-values from multiple comparisons were adjusted using Dunnett’s method after one-way ANOVAs (when comparisons were between a control and all other groups) or Sidak’s method after two-way ANOVAs (when there were more than two groups and planned comparisons). We only performed multiple post hoc tests when statistically significant differences were first observed by ANOVAs. Holm–Sidak’s method was used to adjust comparisons following multiple Student’s *t* tests, Mann–Whitney tests, or Welch’s *t* tests (when there were two groups with multiple genotypes, treatments, or sexes). All statistical tests were two sided. All data represent mean ± SD. Statistical tests were performed using GraphPad Prism V9.1.0.

## Supplementary Material

Appendix 01 (PDF)

## Data Availability

ATAC sequencing data have been submitted to the NCBI Sequence Read Archive, accession number: BioProject PRJNA887449. All study data are included in the article and/or *SI Appendix*.
