# Supplementary material for: Osteolectin increases bone elongation and body length by promoting growth plate chondrocyte proliferation
Source: Proc Natl Acad Sci U S A. 2023 May 22;120(22):e2220159120. doi: 10.1073/pnas.2220159120 (PMC10235998; doi:10.1073/pnas.2220159120)
Supplement: Supplementary file 1 — Appendix 01 (PDF) [file pnas.2220159120.sapp.pdf]

PNAS
www.pnas.org

Supplementary Information for

Osteolectin promotes bone elongation and body lengthening by increasing growth plate chondrocyte proliferation

Jingzhu Zhang, Liming Du, Bethany Davis, Zhimin Gu, Junhua Lyu, Zhiyu Zhao, Jian Xua, Sean J Morrison

Sean J. Morrison
Email: Sean.Morrison@Utsouthwestern.edu

This PDF file includes:

Extended Materials and Methods
Figures S1 to S8

EXTENDED MATERIALS AND METHODS

Genotyping primers

Primers for genotyping *Osteolectin*$^{-/-}$ mice were 5'-GAG GAA GAG GAA ATC ACC ACA, 5'-CGG GAG TCA CAG ATG TTG AA, and 5'- GGC CCA CTG TGA AAA GAC AG. Primers for CRE in *Prx1*^{Cre} and *Aggrecan*^{CreER} mice were 5'-ATG TCC AAT TTA CTG ACC GTA CA and 5'-CGC ATA ACC AGT GAA ACA GCA TT. Primers for *Itga11*^{flox} mice were 5'-AAT TCA GTG CCG ATC CTC CAG TGT C and 5'-CCC TTG CTT CCT TCT GCT GTC ACT T. Primers for *Rosa*$^{loxp\text{-}tdTomato}$ mice were 5'- CTC TGC TGC CTC CTG GCT TCT, 5'- CGA GGC GGA TCA CAA GCA ATA, and 5'- TCA ATG GGC GGG GGT CGT T. Primers for *Osteolectin*^{mTomato} mice were 5'-AAG AGA AGC TGA GGA GAT CAA AAG TTG AGA C, 5'-CCT TAC TTT TCT TTA CCC ATC TGA ACA CTT C, 5'-CAA TTC CGT GGT GTT GTC GGG GAA ATC ATC, and 5'-CAC TGT GAA AAG ACA GAA GGC ACA ACT AGA G.

Immunostaining of bone sections

Freshly dissected mouse femurs were fixed in 4% paraformaldehyde overnight. Bones were decalcified in PBS with 0.5M EDTA for 14 days, followed by 30% sucrose for one day. Bones were sectioned in 10µm slices using the CryoJane system (Leica). Sections were blocked in PBS with 5% normal donkey serum (Jackson ImmunoResearch) for 1 hour and then stained overnight with rabbit-anti-Aggrecan (EMD Millipore, catalog number AB1031), goat-anti-Periostin (R&D Systems, catalog number AF2955), rabbit-anti-tomato (Takara Bio, catalog number 632496), or goat-anti-tomato (LS Bio, catalog number LS-C340696). Donkey-anti-rabbit CF555 (Biotium, catalog number 20038), donkey-anti-rabbit Alexa488 (Jackson ImmunoResearch, catalog number 711-545-152), donkey-anti-goat Alexa488 (Jackson ImmunoResearch, catalog number 705-545-147) or donkey-anti-goat Alexa555 (Invitrogen, catalog number A21432) were used as secondary antibodies. Slides were mounted with Prolong Gold anti-fade plus 4',6-diamidino-2-phenylindole (DAPI; Invitrogen). To label apoptotic cells, the Click-iT Plus TUNEL Assay for In Situ Apoptosis Detection kit (ThermoFisher Scientific) was used. To label dividing cells, mice were given intraperitoneal injections of 5-Ethynyl-2′-deoxyuridine (EdU). P4

mice were injected with a single dose of 0.5mg EdU per mouse and were killed for analysis after 4 hours. P14 mice were injected with a single dose of 1mg EdU per mouse and were killed for analysis after 24 hours. Four week and 8 week-old mice received one injection per day of 1mg EdU per mouse for 2 days and were killed 48 hours after first injection for analysis. EdU was stained using the Click-iT EdU Alexa Fluor 488 Imaging Kit (ThermoFisher Scientific). Images were acquired with a Zeiss LSM780 or LSM880 confocal microscope, and were quantified using ImageJ.

Western blots

Cells were washed with PBS and lysed with RIPA buffer (Sigma Aldrich) plus Halt™ Protease and Phosphatase Inhibitor Cocktail (Thermo Fisher Scientific). The cell lysates were transferred to an Eppendorf tube on ice, incubated for 20 minutes with occasional vortexing, then centrifuged at 16,000xg for 10 minutes at 4°C to clear cellular debris. For analysis of Osteolectin secreted into the culture medium, medium was collected from chondrocyte cultures or hBMSC cultures and centrifuged at 16,000xg for 10 minutes at 4°C then filtered with a 0.20μm syringe to remove cellular debris. The cell lysates or conditioned medium were western blotted with the indicated antibodies and immunoreactive bands were detected using SuperSignal West Dura Extended Duration Substrate or SuperSignal West Femto Maximum Sensitivity Substrate (ThermoFisher Scientific). Antibodies included rabbit-anti-β-catenin (D10A8 clone), rabbit-anti- β-catenin (non-phosphorylated at Ser33/37/Thr41; D13A1 clone), rabbit-anti-β-actin (D6A8 clone) and HRP linked anti-rabbit IgG secondary antibody (catalog number 7074) from Cell Signaling. We also used sheep-anti-human Osteolectin antibody (catalog number AF1904), goat-anti-mouse Osteolectin antibody (catalog number AF3729), HRP linked anti-sheep IgG (catalog number HAF016) and anti-goat IgG (catalog number HAF017) secondary antibodies from R&D Systems. Band intensities were quantitated using ImageJ.

Flow cytometry

To assess cell proliferation in culture, chondrocytes dissected from mice were cultured with 10µM EdU for 24 hours. The chondrocytes were then stained with the Click-iT EdU Alexa Fluor™ 488 Flow Cytometry Assay Kit (Thermofisher Scientific). All samples were analyzed using a FACSAria flow cytometer (BD) and FlowJo v10.7.1 (Tree Star) software.

Reverse transcription quantitative PCR

Cells were washed with PBS and lysed using the Quick-DNA/RNA Miniprep kit (Zymo Research) for DNA/RNA extraction. RNA was reverse transcribed into cDNA using iScript RT (oligo dT and random priming; Bio-Rad). Quantitative PCR (qPCR) was performed using the CFX384 Real-Time System (Bio-Rad). The primers used for qPCR analysis of mouse RNA included: *Actb*: 5'-GCT CTT TTC CAG CCT TCC TT-3' and 5'-CTT CTG CAT CCT GTC AGC AA-3'; *Lef1*: 5'-TGT TTA TCC CAT CAC GGG TGG-3' and 5'-CAT GGA AGT GTC GCC TGA CAG-3'; *Runx2*: 5'-TTA CCT ACA CCC CGC CAG TC-3' and 5'-TGC TGG TCT GGA AGG GTC C-3'; *Alpl*: 5'-CCA ACT CTT TTG TGC CAG AGA-3' and 5'-GGC TAC ATT GGT GTT GAG CTT TT-3'. The primers used for qPCR analysis of human RNA included: *Osteolectin*: 5'- GTG CCA AGA GTC CAG CTT AAT-3' and 5'- ATG GCC AAA GCC CAA GAG-3'; *Actb*: 5'- GGA TCA GCA AGC AGG AGT ATG-3' and 5'- AGA AAG GGT GTA ACG CAA CTA A-3'.

Figure S1

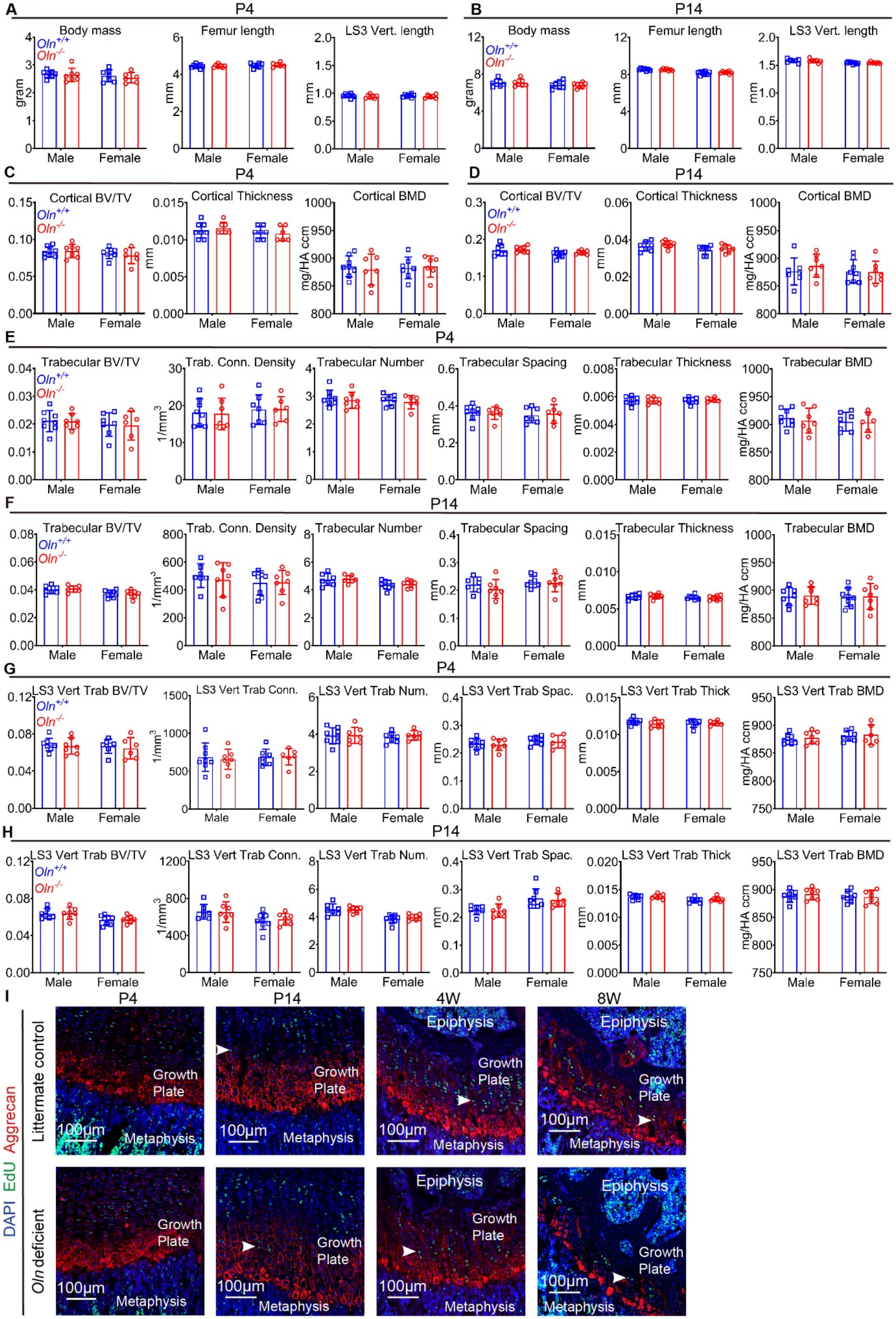

A P4
B P14
Body mass
Femur length
LS3 Vert. length
Oln+/+
Oln-/-
Male Female
C P4
D P14
Cortical BV/TV
Cortical Thickness
Cortical BMD
E P4
F P14
Trabecular BV/TV
Trab. Conn. Density
Trabecular Number
Trabecular Spacing
Trabecular Thickness
Trabecular BMD
G P4
H P14
LS3 Vert Trab BV/TV
LS3 Vert Trab Conn.
LS3 Vert Trab Num.
LS3 Vert Trab Spac.
LS3 Vert Trab Thick
LS3 Vert Trab BMD
I
P4 P14 4W 8W
Littermate control
Oln deficient
DAPI EdU Aggrecan
Epiphysis
Growth Plate
Metaphysis
100μm

Fig. S1. *Osteolectin* deficiency did not affect bone formation or growth prior to 2 weeks of age. (A, B) Body mass, femur length and LS3 vertebra length in *Oln*$^{-/-}$ and sex-matched littermate control (*Oln*$^{+/+}$) mice at P4 (A) or P14 (B). (C, D) MicroCT analysis of cortical bone volume/total volume, cortical thickness, and bone mineral density in the mid-femur diaphysis of *Oln*$^{-/-}$ and *Oln*$^{+/+}$ mice at P4 (C) or P14 (D). (E, F) MicroCT analysis of trabecular bone volume/total volume, connectivity density, number, thickness, spacing, and bone mineral density in the distal femur metaphysis of *Oln*$^{-/-}$ and *Oln*$^{+/+}$ mice at P4 (E) or P14 (F). (G, H) MicroCT analysis of trabecular bone volume/total volume, connectivity density, number, thickness, spacing, and bone mineral density in the third lumbar spine vertebra of *Oln*$^{-/-}$ and *Oln*$^{+/+}$ mice at P4 (G) or P14 (H). Each square/circle represents a different mouse (6-8 mice per sex per age per genotype in 5 independent experiments per age). (I) Representative images of sections from distal femur growth plates of *Oln* deficient (*Oln*$^{-/-}$) and littermate control (*Oln*$^{+/+}$) mice at postnatal day 4 (P4), postnatal day 14 (P14), 4W, and 8W of age. The mice were administered pulses of EdU for 4 hours (P4), 1 day (P14), or 2 days (4W and 8W). DAPI (blue) labels nuclei, Aggrecan (red) labels chondrocytes, and EdU (green) labels dividing cells. Examples of Aggrecan^{+} EdU^{+} cells are indicated by white arrowheads. All statistical tests were two-sided. All data represent mean ± s.d.. Statistical significance was assessed using Student's t-tests followed by Holm-Sidak's multiple comparisons test (Trab. Conn. Density and Trabecular Thickness in E, and LS3 BV/TV in G), or two-way ANOVAs followed by Sidak’s multiple comparisons tests for all other panels.

Figure S2

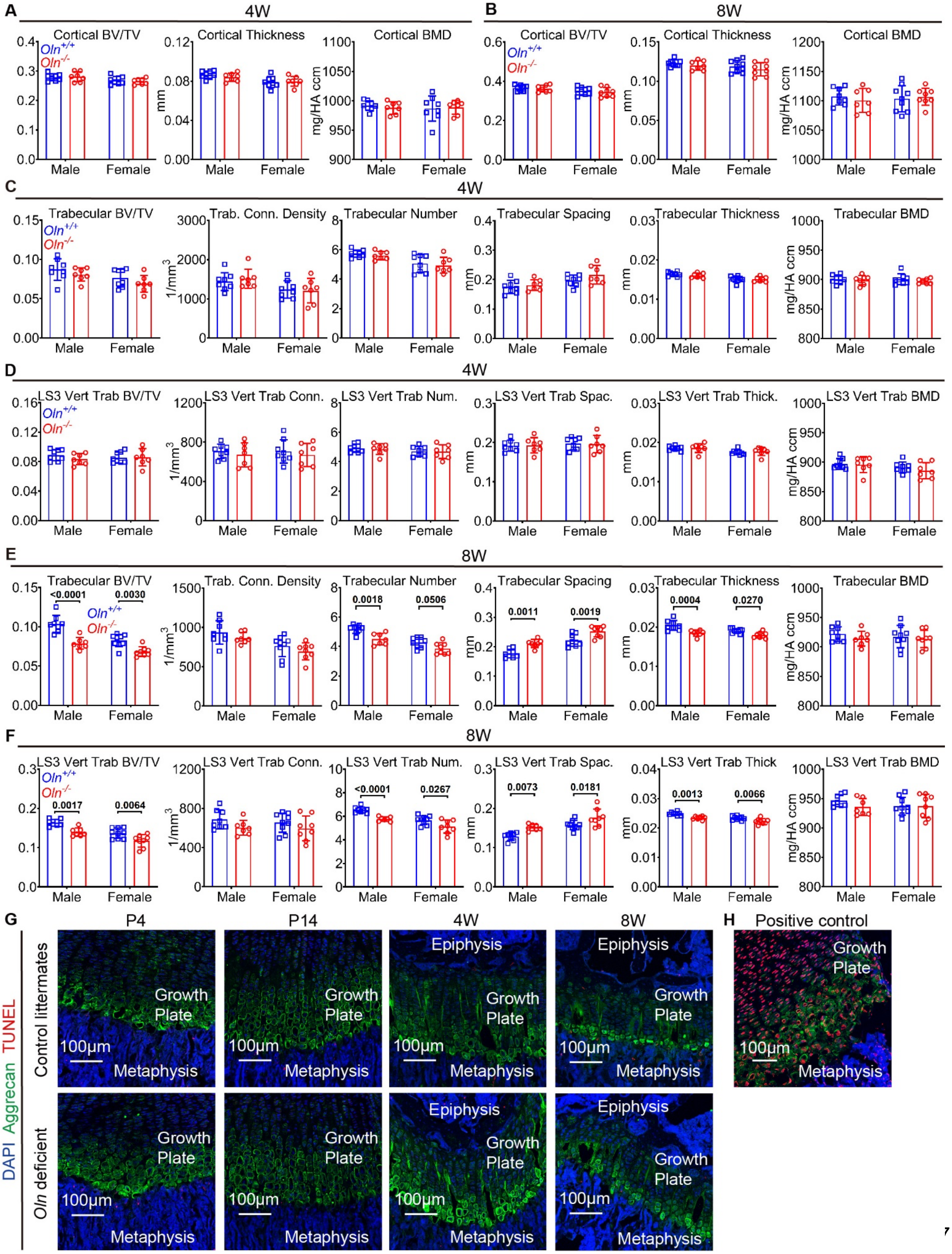

A
4W
Cortical BV/TV
Cortical Thickness
Cortical BMD
B
8W
C
Trabecular BV/TV
Trab. Conn. Density
Trabecular Number
Trabecular Spacing
Trabecular Thickness
Trabecular BMD
D
LS3 Vert Trab BV/TV
LS3 Vert Trab Conn.
LS3 Vert Trab Num.
LS3 Vert Trab Spac.
LS3 Vert Trab Thick.
LS3 Vert Trab BMD
E
F
G
P4
P14
Epiphysis
Growth Plate
Metaphysis
100µm
Control littermates
Oln deficient
DAPI Aggrecan TUNEL
H
Positive control
Male
Female

Fig. S2. *Osteolectin* deficiency reduced trabecular bone at 8 weeks of age. (A, B) MicroCT analysis of cortical bone volume/total volume, thickness, and bone mineral density in the mid-femur diaphysis of *Oln*$^{-/-}$ and sex-matched littermate control (*Oln*$^{+/+}$) mice at 4 (A) or 8 (B) weeks of age. (C-F) MicroCT analysis of trabecular bone volume/total volume, connectivity density, number, thickness, spacing, and bone mineral density in the distal femur metaphysis at 4 weeks of age (C), third lumbar spine vertebra at 4 weeks of age (D), distal femur metaphysis at 8 weeks of age (E), and third lumbar spine vertebra at 8 weeks of age (F). Each circle/square represents a different mouse (7-9 mice per sex per age per genotype in 5 or 6 independent experiments per age). (G) We did not observe any TUNEL (red) stained cells (undergoing cell death) in sections from the distal femur growth plates of *Oln*$^{-/-}$ or control (*Oln*$^{+/+}$) mice at P4, P14, 4 weeks, or 8 weeks of age. (H) As a positive control, a section of P4 *Oln*$^{+/+}$ mouse distal femur growth plate was treated with DNase for 30 minutes to induce DNA damage, and then TUNEL stained. All statistical tests were two-sided. All data represent mean ± s.d.. Statistical significance was assessed using Student's t-tests followed by Holm-Sidak's multiple comparisons tests (Cortical BMD in A, Cortical Thickness in B, Trabecular Number in C, LS3 connectivity and LS3 vertebra thickness in D, and LS3 number in F), and two-way ANOVAs followed by Sidak’s multiple comparisons tests for other panels.

Figure S3

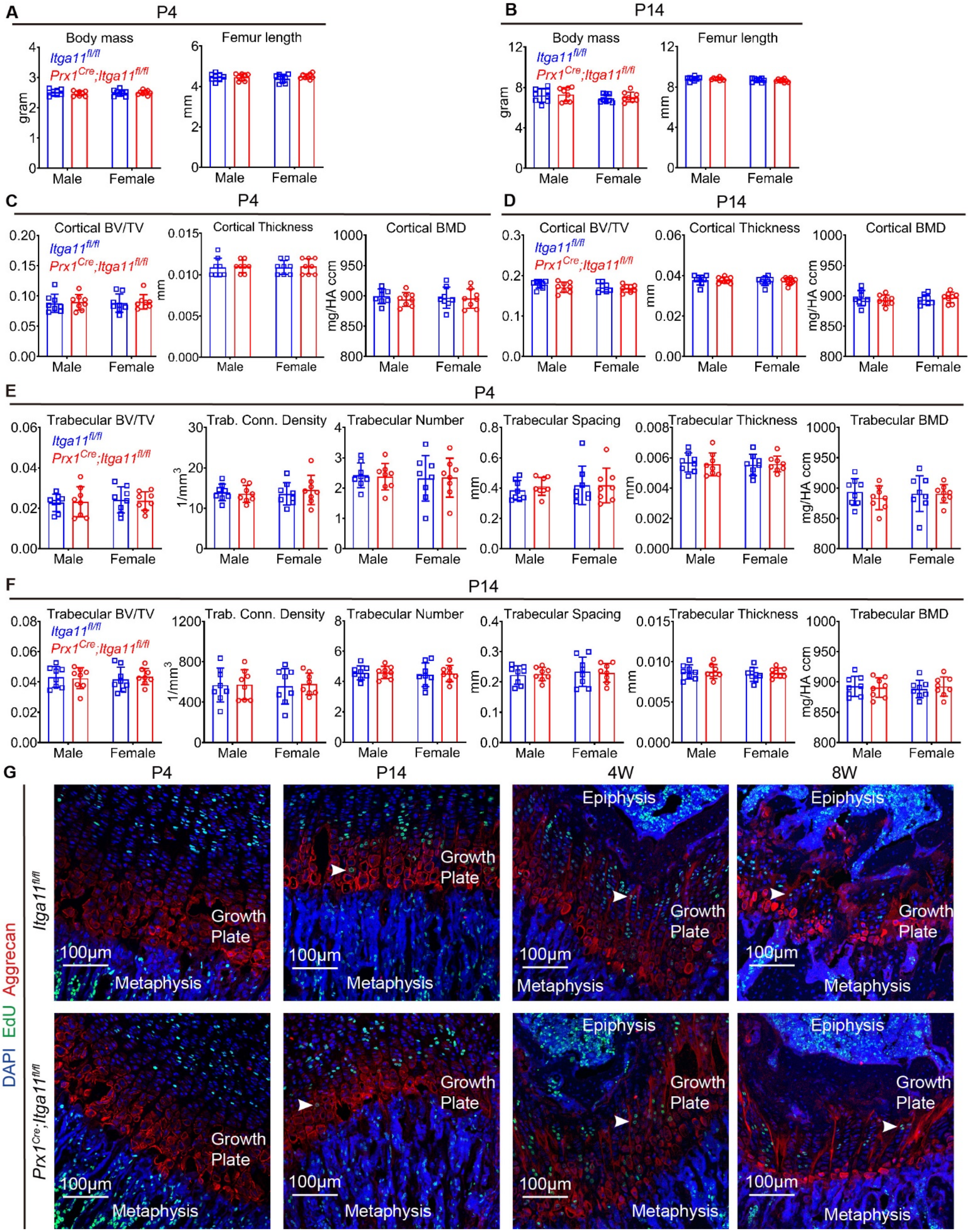
A
P4
Body mass
Femur length
Itga11 fl/fl
Prx1 Cre;Itga11 fl/fl
gram
mm
Male
Female
B
P14
C
Cortical BV/TV
Cortical Thickness
Cortical BMD
mg/HA ccm
D
E
Trabecular BV/TV
Trab. Conn. Density
Trabecular Number
Trabecular Spacing
Trabecular Thickness
Trabecular BMD
1/mm3
F
G
4W
8W
DAPI EdU Aggrecan
Epiphysis
Growth Plate
Metaphysis
100μm

Fig. S3. *Itga11* deficiency in limb mesenchymal cells did not affect bone formation or growth prior to 2 weeks of age. (A, B) Body mass and femur length in $Prx1^{Cre};Itga11^{fl/fl}$ and sex-matched littermate control ($Itga11^{fl/fl}$) mice at P4 (A) and P14 (B). (C, D) MicroCT analysis of cortical bone volume/total volume, thickness, and bone mineral density in the mid-femur diaphysis of $Prx1^{Cre};Itga11^{fl/fl}$ and control mice at P4 (C) and P14 (D). (E, F) MicroCT analysis of trabecular bone volume/total volume, connectivity density, number, spacing, thickness, and bone mineral density in the distal femur metaphysis of $Prx1^{Cre};Itga11^{fl/fl}$ and control mice at P4 (E) and P14 (F). Each square/circle represents a different mouse (8 mice per sex per age per genotype in 5 independent experiments per age). (G) Representative images of sections from distal femur growth plates of $Prx1^{Cre};Itga11^{fl/fl}$ and littermate control mice at P4, P14, 4 weeks and 8 weeks of age. The mice were administered pulses of EdU for 4 hours (P4), 1 day (P14), or 2 days (4W and 8W). All statistical tests were two-sided. All data represent mean ± s.d.. Statistical significance was assessed using Student's t-tests followed by Holm-Sidak's multiple comparisons tests (femur length in A, body mass in B), Mann-Whitney tests followed by Holm-Sidak's multiple comparisons tests (cortical thickness in C), and two-way ANOVAs followed by Sidak’s multiple comparisons tests for all other panels.

Figure S4

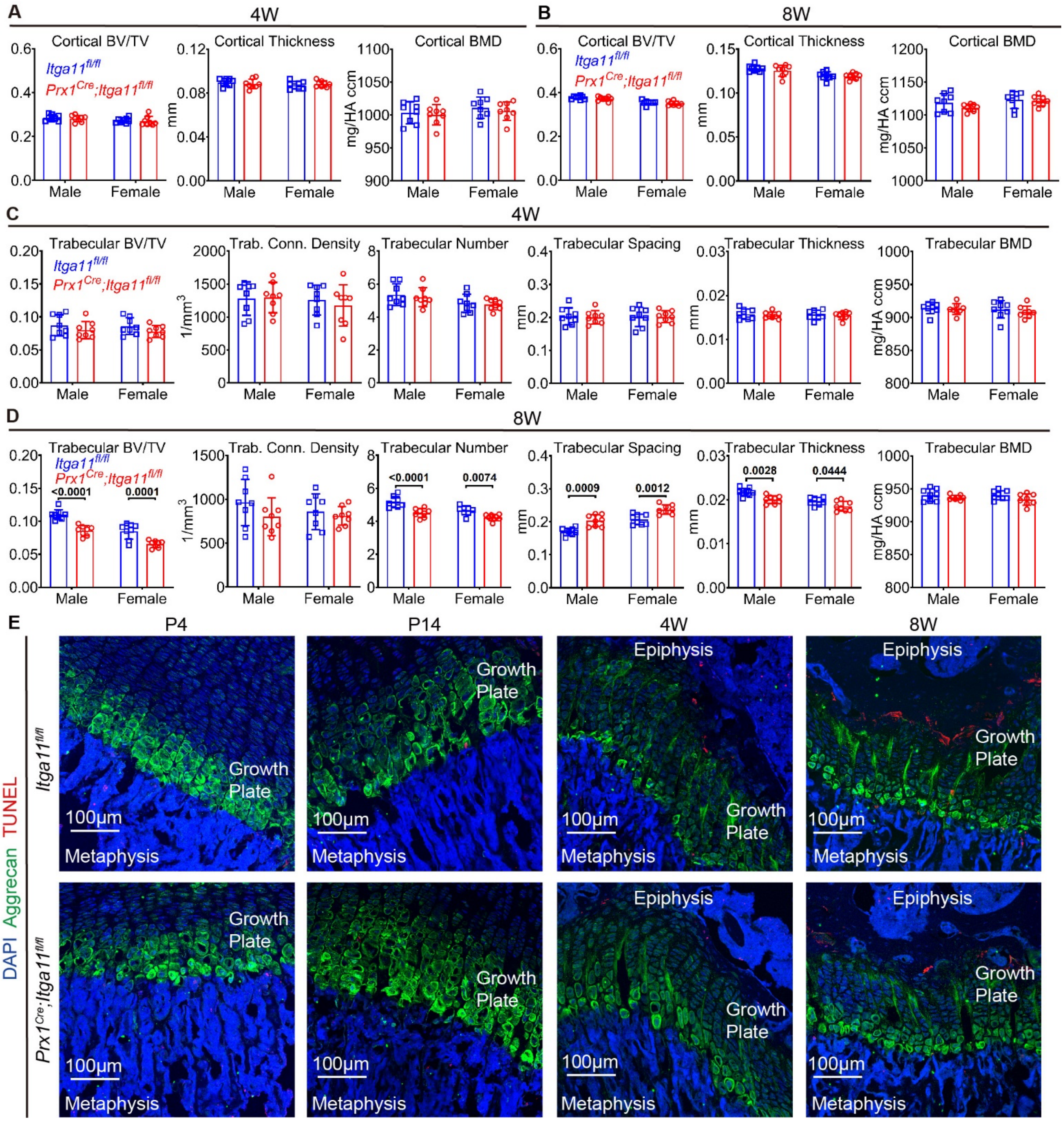

Fig. S4. *Itga11* deficiency in limb mesenchymal cells reduced trabecular bone at 8 weeks of age. (A, B) MicroCT analysis of cortical bone volume/total volume, thickness, and bone mineral density in the mid-femur diaphysis of *Prx1*^{Cre}*;Itga11*$^{fl/fl}$ and sex-matched littermate control (*Itga11*$^{fl/fl}$) mice at 4 (A) and 8 (B) weeks of age. (C, D) MicroCT analysis of trabecular bone volume/total volume, connectivity density,

number, spacing, thickness, and bone mineral density in the distal femur of *Prx1^{Cre};Itga11$^{fl/fl}$* and *Itga11$^{fl/fl}$* mice at 4 (C) and 8 (D) weeks of age. Each square/circle represents a different mouse (8 mice per sex per age per genotype in 5 independent experiments per age). (E) Representative images of sections from femur growth plates of *Prx1^{Cre};Itga11$^{fl/fl}$* and sex-matched littermate control (*Itga11$^{fl/fl}$*) mice at P4, P14, 4 weeks and 8 weeks of age. All statistical tests were two-sided. All data represent mean ± s.d.. Statistical significance was assessed using Student's t-tests followed by Holm-Sidak's multiple comparisons tests (trabecular BV/TV in C, trabecular spacing in D) or two-way ANOVAs followed by Sidak’s multiple comparisons tests for other panels.

Figure S5

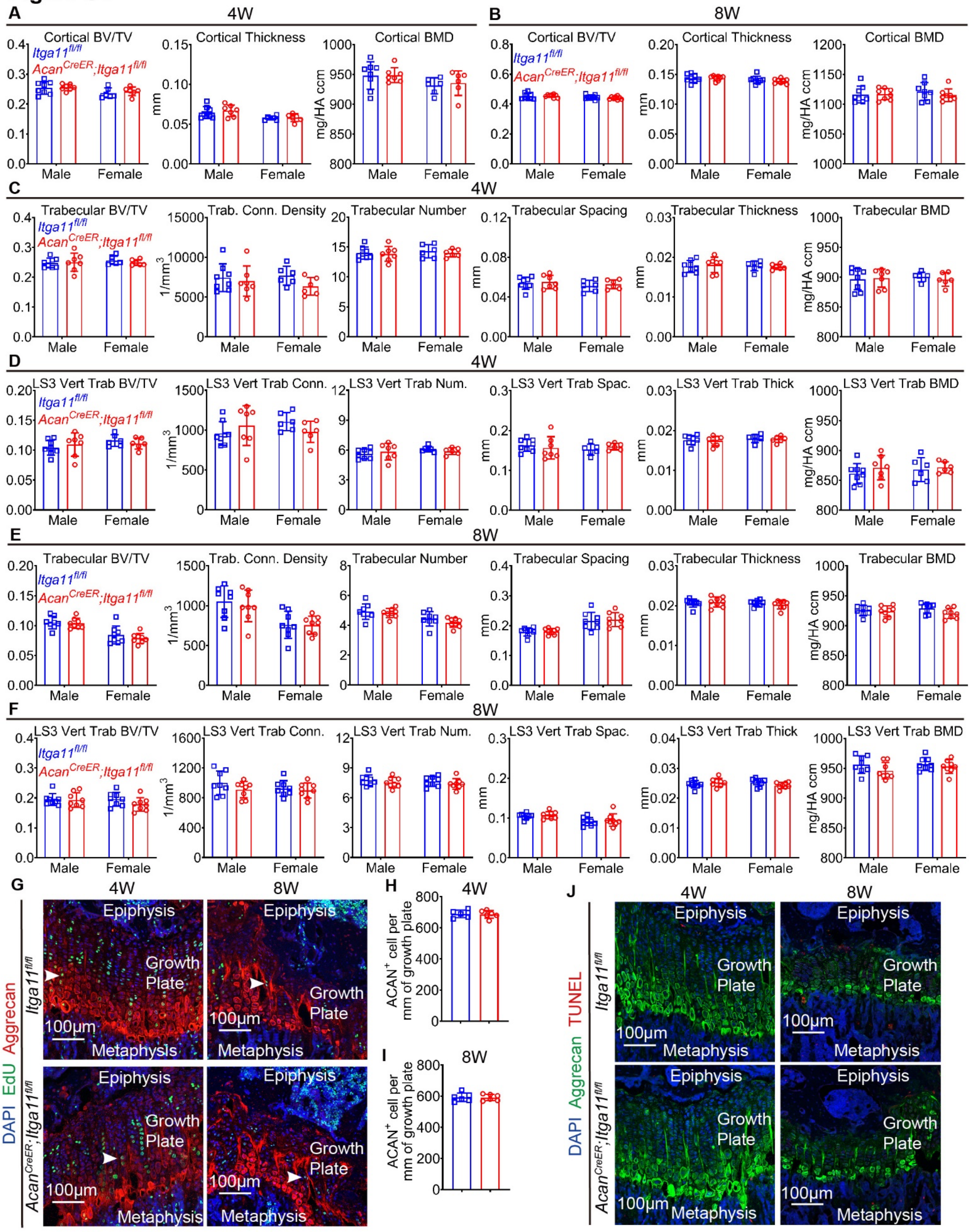
A
4W
Cortical BV/TV
Cortical Thickness
Cortical BMD
B
8W
C
Trabecular BV/TV
Trab. Conn. Density
Trabecular Number
Trabecular Spacing
Trabecular Thickness
Trabecular BMD
D
LS3 Vert Trab BV/TV
LS3 Vert Trab Conn.
LS3 Vert Trab Num.
LS3 Vert Trab Spac.
LS3 Vert Trab Thick
LS3 Vert Trab BMD
E
F
Itga11fl/fl
AcanCreER;Itga11fl/fl
Male
Female
mm
mg/HA ccm
1/mm3
G
H
I
J
DAPI EdU Aggrecan
DAPI Aggrecan TUNEL
Epiphysis
Growth Plate
Metaphysis
100μm
ACAN+ cell per mm of growth plate

Fig. S5. *Acan*^{CreER};*Itga11*$^{fl/fl}$ and sex-matched littermate control (*Itga11*$^{fl/fl}$) mice were treated with tamoxifen at 2 weeks of age and then cortical and trabecular bone parameters were analyzed at 4 or 8 weeks of age. (A, B) MicroCT analysis of cortical bone volume/total volume, thickness, and bone mineral density in the mid-femur diaphysis at 4 (A) and 8 (B) weeks of age. (C, D) MicroCT analysis of trabecular bone volume/total volume, connectivity density, number, thickness, spacing, and bone mineral density in the distal femur metaphysis (c) or in the third lumbar spine vertebra (D) at 4 weeks of age. (E, F) MicroCT analysis of trabecular bone volume/total volume, connectivity density, number, thickness, spacing, and bone mineral density in the distal femur metaphysis (E) or in the third lumbar spine vertebra (F) at 8 weeks of age. Each square/circle represents a different mouse (6-8 mice per sex per age per genotype in 4 or 5 independent experiments per age). (G) Representative images of sections from distal femur growth plates of *Acan*^{CreER};*Itga11*$^{fl/fl}$ and control mice administered a 2 day pulse of EdU at 4 weeks and 8 weeks of age. (H, I) Numbers of Aggrecan^{+} chondrocytes per mm of growth plate in *Acan*^{CreER};*Itga11*$^{fl/fl}$ and control mice at 4 (H) or 8 (I) weeks of age (3 mice per sex per age per genotype in 4 or 3 independent experiments per age). (J) We did not observe any TUNEL (red) stained cells in femur growth plate sections from *Acan*^{CreER};*Itga11*$^{fl/fl}$ and control mice at 4 or 8 weeks of age. All statistical tests were two-sided. All data represent mean ± s.d.. Statistical significance was assessed using Student's t-tests followed by Holm-Sidak's multiple comparisons tests (cortical thickness in A, trabecular thickness in C, LS3 number in D, H and I), or two-way ANOVAs followed by Sidak’s multiple comparisons tests for other panels.

Figure S6

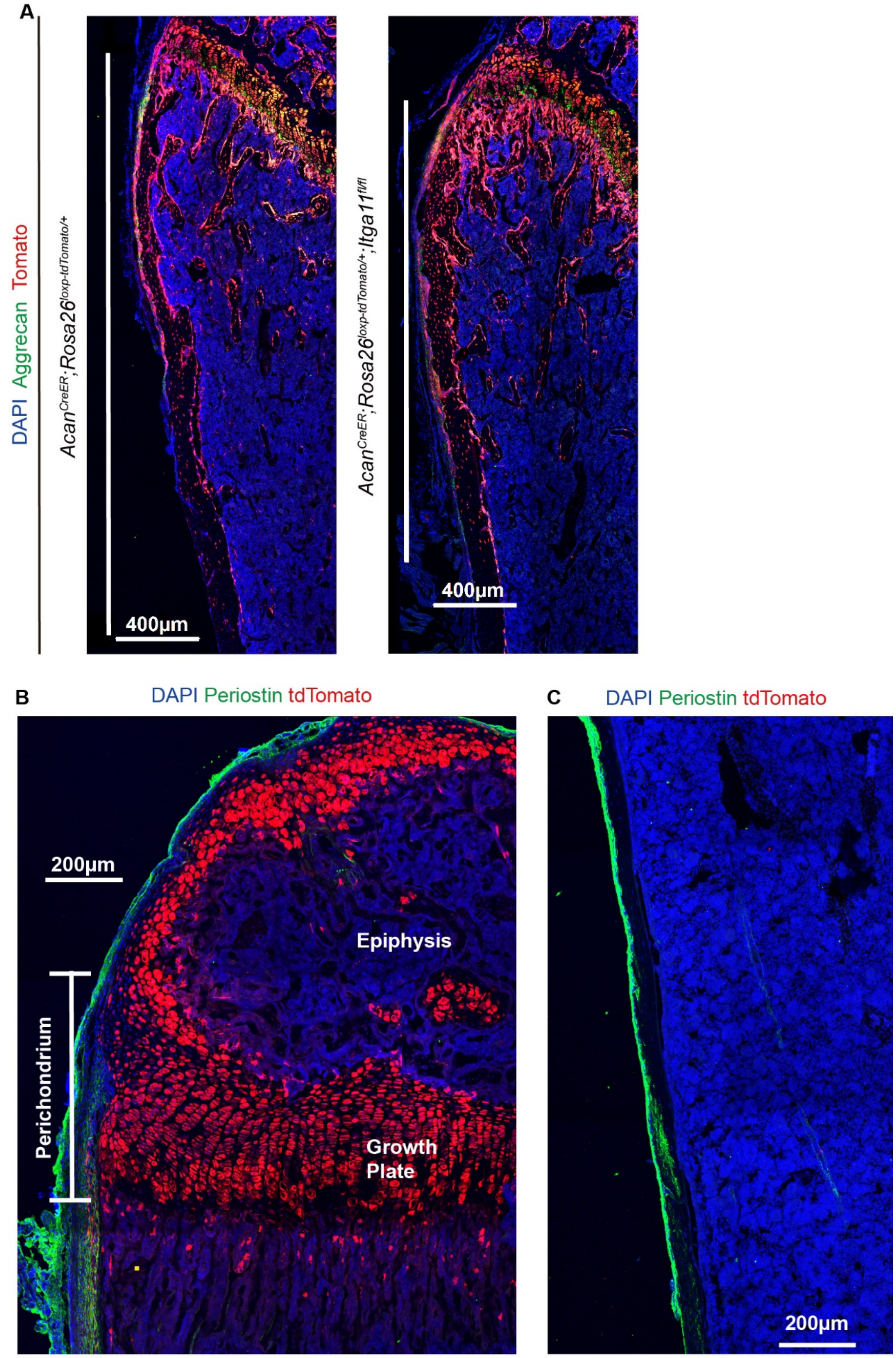
A
DAPI Aggrecan Tomato
Acan^CreER;Rosa26^loxp-tdTomato/+
Acan^CreER;Rosa26^loxp-tdTomato/+;Itga11^fl/fl
400μm
400μm
B
DAPI Periostin tdTomato
200μm
Epiphysis
Perichondrium
Growth
Plate
C
DAPI Periostin tdTomato
200μm

Fig. S6. Lineage tracing of cortical bone that arose from growth plate chondrocytes. (A) Representative images of femur sections from 8-week old *Acan*^{CreER}*;Rosa26*$^{loxp\text{-}tdTomato/+}$*;Itga11*$^{fl/fl}$ and sex-matched littermate control (*Acan*^{CreER}*;Rosa26*$^{loxp\text{-}tdTomato/+}$) mice that were treated with tamoxifen at 2 weeks of age. In these images, Aggrecan (green) labels chondrocytes and Tomato (red) labels cells that arose from chondrocytes. The white lines showed the length of cortical bone that arose from chondrocytes since tamoxifen treatment. (B, C) Acan-CreER recombines in very few perichondrial cells at 2 weeks of age. Representative image of femur growth plates (B) and diaphysis (C) from 2-week old *Acan*^{CreER}*;Rosa26*$^{loxp\text{-}tdTomato/+}$ mice that were treated with 1 dose of tamoxifen and harvested 2 days later, DAPI marks nuclei, Tomato marks cells in which Acan-CreER recombined (or cells they gave rise to), and periostin marks perichondrial cells.

Figure S7

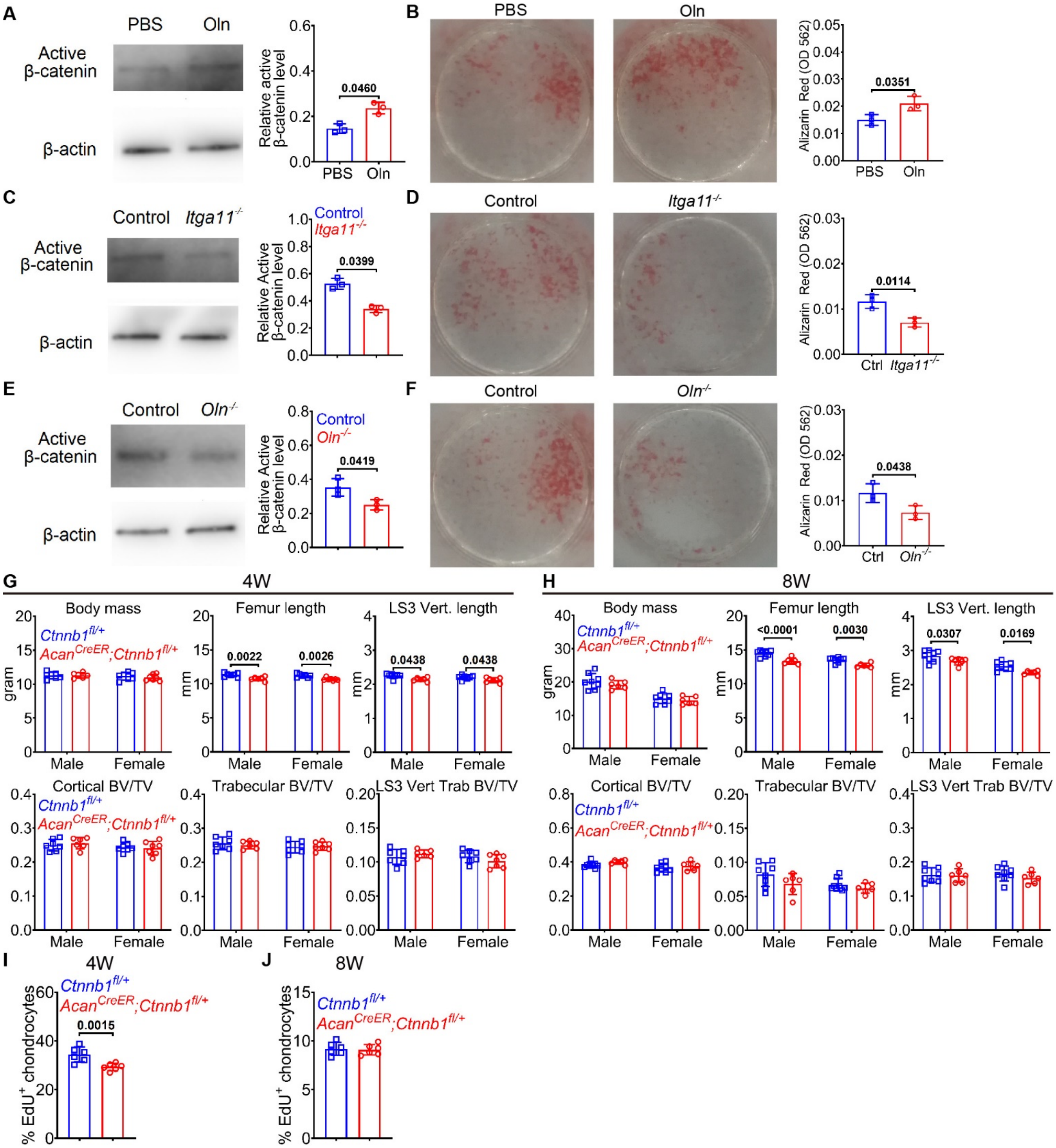

Fig. S7. Osteolectin/Integrin α11 signaling promotes Wnt pathway activation and the osteogenic differentiation of growth plate chondrocytes. (A-B) Growth plate chondrocytes from 4 week-old male wild-type mice were cultured with Osteolectin (Oln) or PBS for 2 days. Active β-catenin (unphosphorylated at GSK3-dependent sites) levels were quantified by western blot (A). These

chondrocytes were then cultured in osteogenic differentiation medium for 3 weeks with Osteolectin or PBS, and osteogenic differentiation was assessed by Alaizarin Red staining (B). (C-D) Growth plate chondrocytes from 4 week-old male *Acan*^{CreER}*;Itga11*$^{fl/fl}$ and littermate *Itga11*$^{fl/fl}$ control mice were cultured with 4-hydroxytamoxifen for 2 days to delete Integrin α11. Active β-catenin levels were quantified by western blot (C). The osteogenic differentiation of these chondrocytes was assessed by culturing in osteogenic differentiation medium for 3 weeks and staining with Alizarin Red (D). (E-F) Growth plate chondrocytes from 4 week-old male *Oln*$^{-/-}$ and littermate control mice were cultured for 2 days. Active β-catenin levels were quantified by western blot (E). The osteogenic differentiation of these chondrocytes was then assessed by culturing in osteogenic differentiation medium for 3 weeks and staining with Alizarin Red (F) (3 mice per genotype in 3 independent experiments for A-F). (G, H) *Acan*^{CreER}*;Ctnnb1*$^{fl/+}$ and sex-matched littermate control (*Ctnnb1*$^{fl/+}$) mice were treated with tamoxifen at 2 weeks of age, then body mass, femur length, LS3 vertebra length, femur cortical bone volume, and trabecular bone volume in femurs and vertebrae were measured at 4 weeks (G) and 8 weeks (H) of age. Each square/circle represents a different mouse (6-8 mice per sex per age per genotype in 4 or 5 independent experiments per age). (I, J) The percentages of Aggrecan^{+} growth plate chondrocytes that incorporated a 2 day pulse of EdU at 4 (I) or 8 (J) weeks of age (3 mice per sex per genotype per age in 3 independent experiments per age). All statistical tests were two-sided. All data represent mean ± s.d.. Statistical significance was assessed using paired t-tests (A-F), and Mann-Whitney tests followed by Holm-Sidak's multiple comparisons tests (LS3 Vert. Length in G), and Student's t-tests followed by Holm-Sidak's multiple comparisons test (LS3 Trab BV/TV in G and Trabecular BV/TV in H), and two-way ANOVAs followed by Sidak’s multiple comparisons tests (other panels in G and H), or Student's t-tests followed by Holm-Sidak's multiple comparisons tests (I, J).

Figure S8

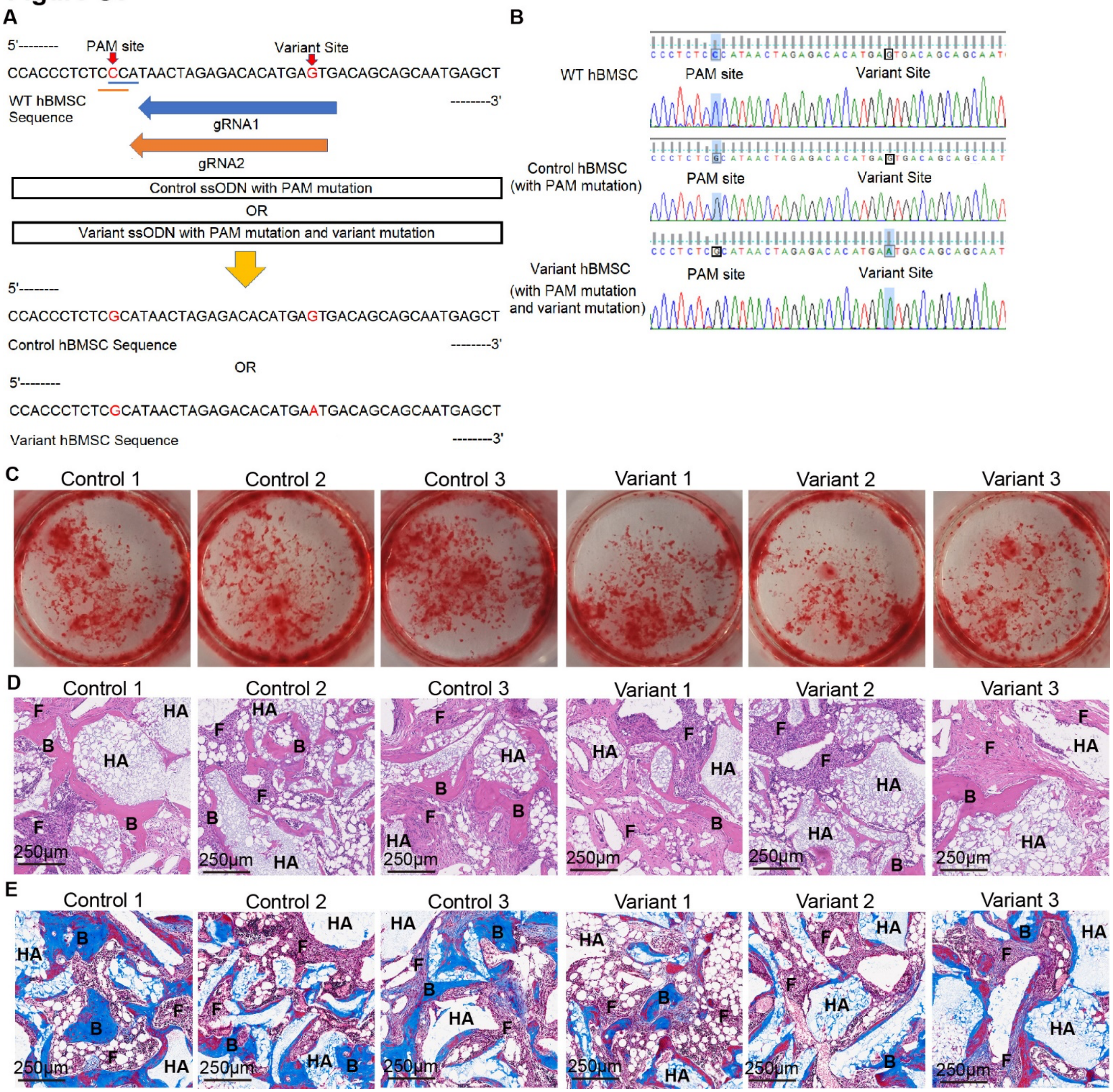

Fig. S8. The rs182722517 variant reduces osteogenic differentiation by human bone marrow stromal cells (hBMSCs). (A) Strategy for CRISPR editing of primary hBMSCs to introduce homozygous or heterozygous rs182722517 variants. Two guide RNAs (gRNA1 and gRNA2) were used to cut the wild-type sequence and single-stranded oligodeoxynucleotides (ssODNs) were added to introduce the variant. The control ssODN had a C>G mutation in the PAM site that increased editing efficiency by preventing re-cutting. The variant ssODN had this C>G mutation in the PAM site as well as a G>A

mutation at the rs182722517 variant site. (B) Representative Sanger sequencing shows the wild-type sequence, the control (homozygous PAM site mutations only) sequence, and the rs182722517 variant (homozygous PAM site and variant mutations) sequence. (C) Representative images of Alizarin Red staining in homozygous control or rs182722517 variant clones after culture for 21 days in osteogenic differentiation medium. (D-E) Representative images of hematoxylin and eosin staining (D) and trichrome staining (E) of boney ossicles formed by homozygous control or rs182722517 variant hBMSCs 6 weeks after subcutaneous transplantation into NSG mice (B represents bone, F represents fibroblasts, and HA represents hydroxyapatite/tricalcium phosphate particles).
